# YAP Inactivation by Soft Mechanotransduction Relieves MAFG for Tumor Cell Dedifferentiation

**DOI:** 10.34133/research.0215

**Published:** 2023-08-22

**Authors:** Jiadi Lv, Xiaohan Liu, Yabo Zhou, Feiran Cheng, Haoran Chen, Shunshun Li, Dianheng Wang, Li Zhou, Zhenfeng Wang, Nannan Zhou, Jie Chen, Bo Huang

**Affiliations:** ^1^ Department of Immunology & State Key Laboratory of Common Mechanism Research for Major Diseases, Institute of Basic Medical Sciences, Chinese Academy of Medical Sciences & Peking Union Medical College (PUMC), Beijing, 100005, China.; ^2^ Department of Histology and Embryology, Basic Medical College, China Medical University, Shenyang, Liaoning 110122, China.; ^3^ Department of Immunology, Basic Medical College, China Medical University, Shenyang, Liaoning 110122, China.; ^4^ Department of Biochemistry & Molecular Biology, Tongji Medical College, Huazhong University of Science & Technology, Wuhan 430030, China.

## Abstract

Solid tumor cells live in a highly dynamic mechanical microenvironment. How the extracellular-matrix-generated mechanotransduction regulates tumor cell development and differentiation remains an enigma. Here, we show that a low mechanical force generated from the soft matrix induces dedifferentiation of moderately stiff tumor cells to soft stem-cell-like cells. Mechanistically, integrin β8 was identified to transduce mechano-signaling to trigger tumor cell dedifferentiation by recruiting RhoGDI1 to inactivate RhoA and subsequently Yes-associated protein (YAP). YAP inactivation relieved the inhibition of v-maf avian musculoaponeurotic fibrosarcoma oncogene homolog G (MAFG), allowing MAFG to transactivate the stemness genes *NANOG*, *SOX2*, and *NESTIN*. Inactivation also restored β8 expression, thereby forming a closed mechanical loop. Importantly, MAFG expression is correlated with worse prognosis. Our findings provide mechanical insights into the regulation of tumor cell dedifferentiation, which has therapeutic implications for exploring innovative strategies to attack malignancies.

## Introduction

Cancer stem cells (CSCs), a notion based on the observation that only a very small population of cells from a tumor can seed and form a tumor in immunodeficient mice, represent undifferentiated tumor cells that survive in harsh microenvironments, metastasize to distant organs, evade immune attack or enter a dormant state, and resist various treatments. Thus, targeting CSCs may hold promise in preventing disease progression and efficiently eradicating cancer, which, however, calls for a deeper comprehension of the origin of CSCs. Recent research has shown that differentiated non-stem cancer cells can be converted into undifferentiated CSCs in human breast tumors [1,2]. The existence of tumor cell dedifferentiation greatly complicates approaches to targeting CSCs, but the underlying mechanism remains elusive.

Stiffness is a mechanical property of a cell, which is mainly contributed by F-actin filaments [3,4]. Different cell types display various levels of stiffness in order to match the stiffness of local tissues, thus allowing cells to properly sense and respond to surrounding mechanical microenvironment [5,6]. Cell stiffness can range from 0.01 kPa for unfertilized egg cells [7], 0.5 kPa for embryonic stem cells [8], 1 to 5 kPa for differentiated tissue cells [9], to 12 kPa for skeletal muscle cells [10]. Thus, cellular stiffness appears to be related to the differentiation state of cells, and a weak stiffness represents a less differentiated state. In line with this concept, undifferentiated tumorigenic cells are much softer (soft is the inverse of stiff) than their differentiated counterparts in various cancer types [11]. Based on this understanding, we hypothesize that the dedifferentiation of tumor cells is regulated by mechanical signaling generated from a soft mechanical microenvironment. Previously, we developed a mechanics-based culture system employing 3-dimensional (3D) soft fibrin gels to amplify highly tumorigenic cells that may repopulate tumors (tumor-repopulating cells [TRCs]) from cancer cell lines and patients’ tumor tissues [12–17]. These TRCs amplified by the 3D gel are soft and exhibit conventional traits of CSCs, such as CD133^+^ or ALDH^+^ and the ability to generate a tumor from as few as 5 or 1 cell [13]. Therefore, in this study, we adopt this method and provide evidence that partially differentiated breast and melanoma cancer cells use integrin β8 to sense a low mechanical force, which inactivates Yes-associated protein (YAP) and relieves its suppression of v-maf avian musculoaponeurotic fibrosarcoma oncogene homolog G (MAFG), leading to transactivating stemness genes and tumor cell dedifferentiation.

## Results

### Tumor cell dedifferentiation occurs in a soft mechanical microenvironment

**Fig. 1.**
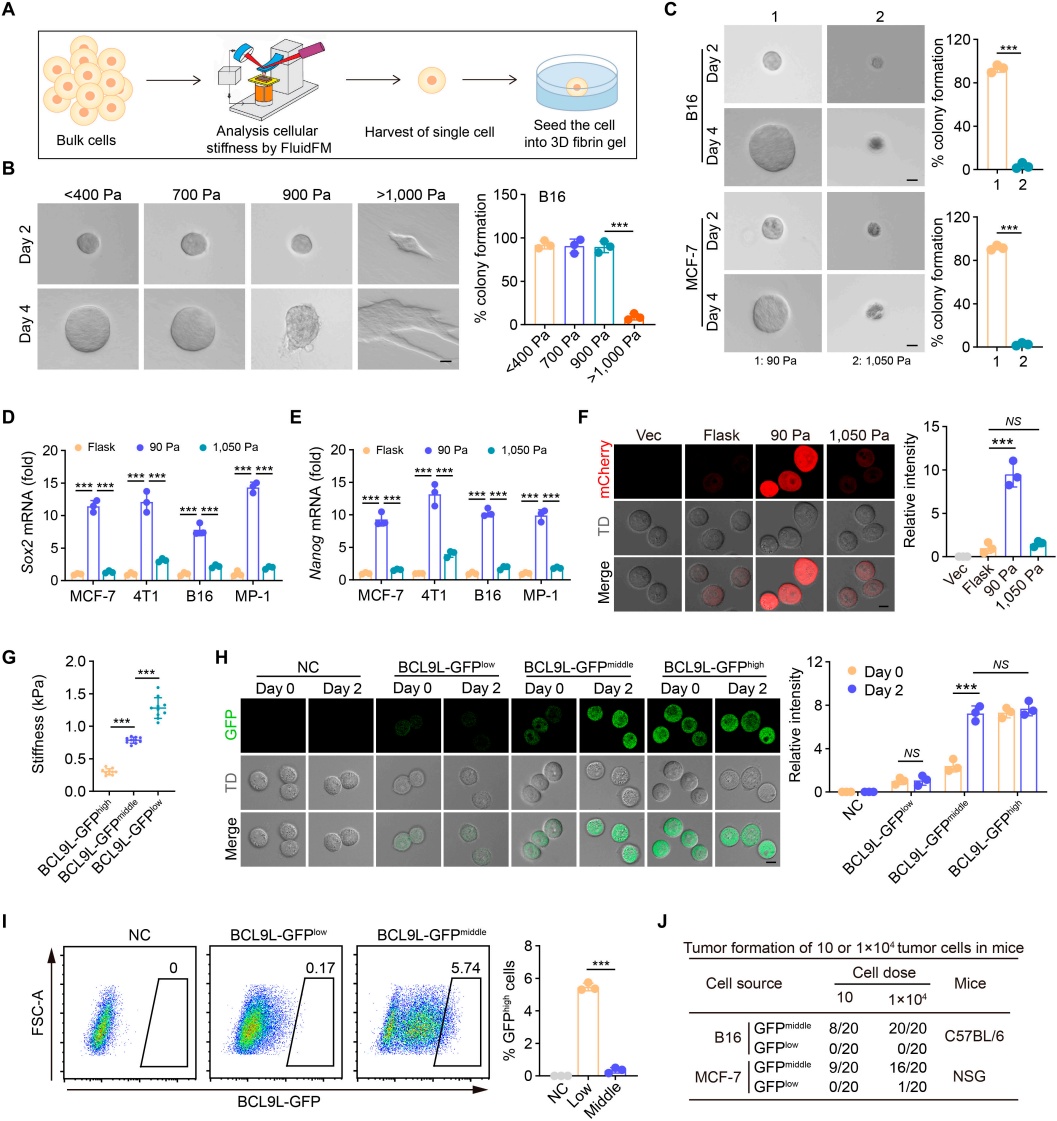
The soft mechanical microenvironment could induce tumor cell dedifferentiation. (A) Schema of experimental design. Briefly, the stiffness of tumor cells was analyzed by atomic force microscopy (AFM). Then, the cells were harvested and seeded into the 3D fibrin gels. (B) The B16 cells with a different stiffness (100 cells per group) were seeded in the 90-Pa soft 3D fibrin gel for 4 days. The percentage of colony formation was calculated. Scale bar, 20 μm. (C) The M-stiff tumor cells were seeded in the 90-Pa or 1,050-Pa 3D fibrin gel for 4 days. The colonies were presented photographically (left) and the percentage of colony formation was calculated (right). Scale bar, 20 μm. (D and E) The mRNA expression of *Sox2* (D) and *Nanog* (E) in B16, 4T1, MCF-7, or MP1 cells cultured in the 90-Pa or 1,050-Pa soft 3D fibrin gel or on a rigid plate (flask) for 48 h was detected by qPCR. (F) The relative intensity of mCherry in *Sox2* promoter-mCherry-expressing B16 cells cultured on a rigid plate or in the 90-Pa or 1,050-Pa 3D fibrin gel for 48 h was observed under a confocal microscope. Scale bar, 10 μm. (G) The stiffness of GFP^low^, GFP^mid^, or GFP^high^ tumor cells from *BCL9L* promoter-GFP-expressing MCF-7 was measured by FluidFM. *n* = 10. (H) H-stiff, M-stiff, and soft cells were cultured in the 90-Pa 3D fibrin gel for 48 h. The expression of GFP in *BCL9L* promoter-GFP-expressing different stiffness MCF-7 cells at 0 h and 48 h was observed under a confocal microscope. Scale bar, 10 μm. (I) A total of 1 × 10^6^ BCL9L-GFP^low^ and GFP^middle^ tumor cells were sorted from *BCL9L* promoter-GFP-expressing MCF-7 cells and then subcutaneously inoculated into NSG mice, respectively. Three days later, the expression of GFP in a single tumor cell was analyzed by flow cytometry. *n* = 3. (J) GFP^low^ or GFP^middle^ tumor cells from *BCL9L* promoter-GFP-expressing B16 or MCF-7 cells embedded in 50 μl of fibrin gel (90 Pa) were inserted into the subcutaneous site of C57BL/6 or NSG mice (*n* = 20). The tumor formation was recorded. In (B) to (E) and (I), *n* = 3 biological independent experiments. ***P < 0.001, by 2-tailed Student’s *t* test (C and H) or one-way ANOVA Bonferroni’s test (B, D to G, and I). *NS*, no significant difference. The data represent mean ± SD.

Although collagen is the main component of tumor extracellular matrices, fibrin also exists ubiquitously within tumors [18–20]. Intriguingly, the levels of fibrinogen were correlated with poor prognosis of patients with different cancer types (Fig. S1A to D), suggesting that the fibrin matrix profoundly affects tumor cell behavior. Previously, we demonstrated that tumorigenic cells are able to grow spheroidic colonies in soft 3D fibrin gels (90 Pa) and the amplified progeny cells termed TRCs, which can repopulate tumors [12–17]. Such TRCs display mechanical softness (<400 Pa); in contrast, differentiated tumor cells are stiff [11], implying that a soft mechanical microenvironment might promote tumor cell dedifferentiation, considering the consistency of cell stiffness with tissue stiffness [21,22]. In line with this notion, single B16 melanoma cells with a stiffness >1,000 Pa, which were picked up via fluidic force microscopy (FluidFM), did not form spheroids when in 90-Pa soft 3D fibrin gels (Fig. 1A and B). Surprisingly, cells with stiffness between 700 and 900 Pa could grow colonies in the 90-Pa gel, similar to the less than 400-Pa soft TRCs (Fig. 1B). Consistent results were obtained from MCF-7 human breast cancer cells (Fig. S1E). Notably, seeding the ~700-Pa cells into moderately stiff 1,050-Pa fibrin gels did not form spheroid colony and likely underwent death (Fig. 1C). Given that softness represents stem-cell-like cancer cells [11,16,23], the above results suggest that the highly stiff cells (H-stiff, ≥1,000 Pa), which are likely to differentiate well, cannot dedifferentiate into soft tumorigenic cells; however, tumor cells with moderate stiffness (M-stiff, 700 to 900 Pa), a state of moderate differentiation, have the potential to dedifferentiate. Sox2, Nanog, and Nestin are typical stem cell markers for B16 and MCF-7, and were upregulated in the dedifferentiated spheroid cells (Fig. 1D and E and Fig. S1F). We constructed *Sox2* promoter-mCherry-expressing B16 cells. Red mCherry, although not expressed in M-stiff cells, was induced in the 90-Pa fibrin gels, but not in the 1,050-Pa fibrin gels (Fig. 1F). In addition to Sox2, microphthalmia-associated transcription factor (MITF) is a marker of melanoma cell differentiation [24]. Consistently, M-stiff melanoma cells downregulated MITF upon seeding in soft gels, in which H-stiff cells did not alter the high expression of MITF (Fig. S1G and H). To further verify this dedifferentiation pattern, we constructed *BCL9L* promoter-GFP-expressing MCF-7 cells given that BCL9L is strongly expressed in soft TRCs but very weakly expressed in stiff cells [11]. As measured by FluidFM, the sorted GFP^low^, GFP^middle^, and GFP^high^ cells had the corresponding stiffness of 1,000 to 1,600 Pa, 700 to 900 Pa, and <400 Pa, thus representing H-stiff, M-stiff, and soft cells, respectively (Fig. 1G). As expected, M-stiff rather than H-stiff cells could be converted to GFP^high^ soft cells in 90-Pa fibrin gels (Fig. 1H). These in vitro results could be validated in vivo by inoculating 1 × 10^6^ M-stiff and H-stiff cells into mice for 3 days. We found that GFP^high^ soft tumor cells were not found in the H-stiff group; however, they were formed in the M-stiff group at a ratio of 5.74% (Fig. 1I). Following the isolation of the 5.74% cells, 100 such cells could form a tumor in NSG mice with a proportion of 7/20 (Fig. S1I), indicating that M-stiff tumor cells are indeed able to dedifferentiate into stem-cell-like cancer cells. In line with this, inserting a piece of 90-Pa gel, which contained 10 M-stiff cells within 50 μl volume, into a subcutaneous site of the mice, the tumor formation appeared in mice (8/20) in 8 weeks (Fig. 1J). However, the inoculation of 10^4^ H-stiff cells could not form a tumor in the mice (Fig. 1J). In addition, the serial transplantation assay showing tumor formation from each generation of 100 GFP^high^ cells (Fig. S1J) confirmed that the GFP^high^ cells possessed self-renewing capacity. Together, these results suggest that M-stiff tumor cells can dedifferentiate to soft stem-cell-like tumor cells in a soft mechanical microenvironment.

### Integrin β8 mediates mechanotransduction for the dedifferentiation

**Fig. 2.**
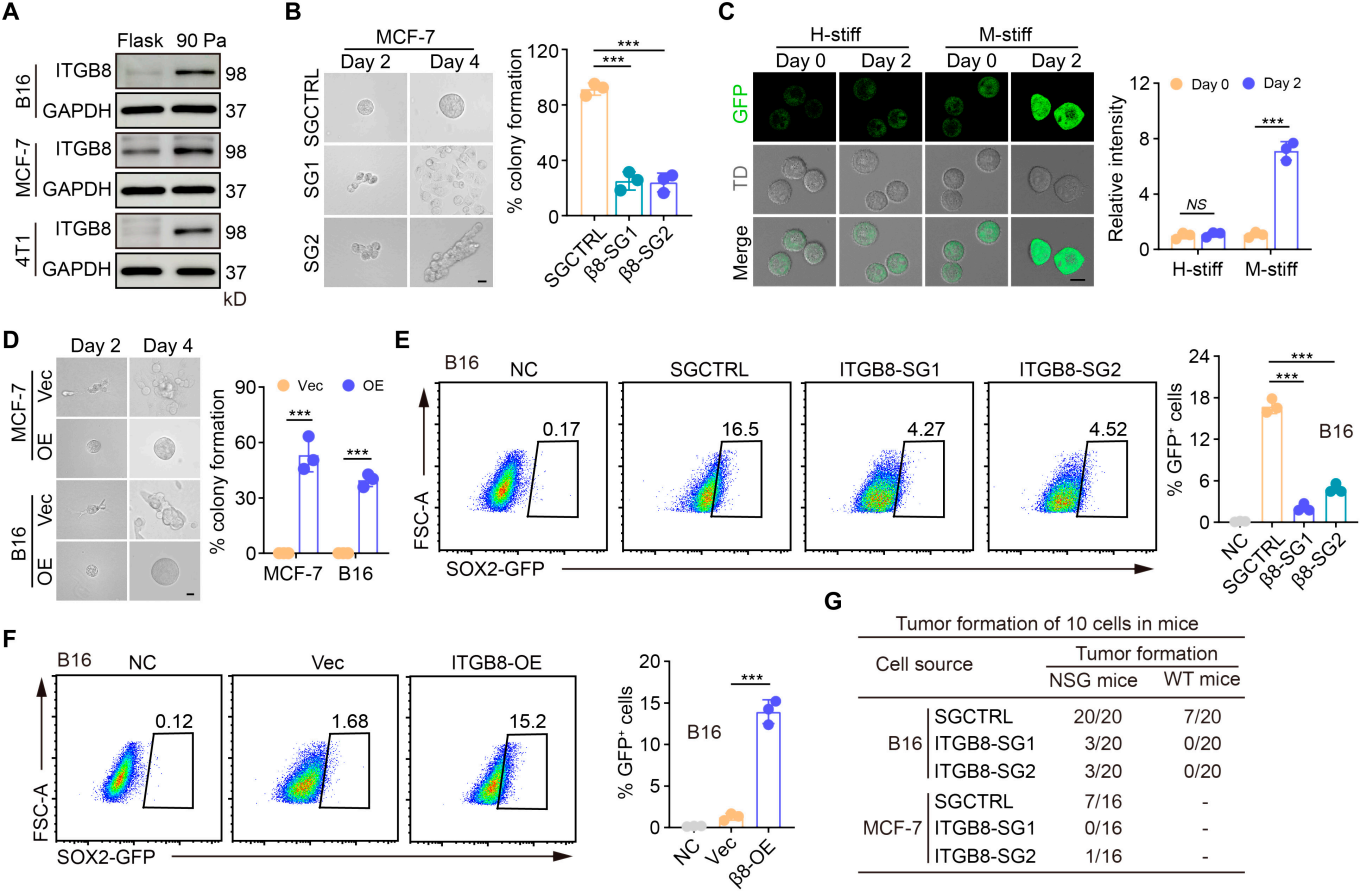
Integrin β8 mediates the mechanotransduction for tumor cell dedifferentiation. (A) The level of ITGB8 in M-stiff B16, 4T1, or MCF-7 tumor cells cultured in the 90-Pa soft 3D fibrin gel or flask for 48 h was analyzed by Western blot. (B) SGCTRL or *ITGB8*-SGs-MCF-7 cells were seeded in the 90-Pa soft 3D fibrin gel for 4 days. The colonies were presented photographically (left) at day 2 or day 4 and the percentage of colony formation was calculated (right). Scale bar, 20 μm. (C) The expression of GFP in H-stiff or M-stiff *ITGB8* promoter-GFP-expressing MCF-7 cells cultured in the 90-Pa 3D fibrin gel at day 2 or day 4 was observed under a confocal microscope. Scale bar, 10 μm. (D) Same as (B), but for H-stiff vector or *ITGB8*-OE-B16 or MCF7 cells. Scale bar, 20 μm. (E) A total of 1 × 10^6^ M-stiff SGCTRL or *Itgb8*-SGs-B16 cells, which were Sox2 promoter-GFP-expressing, were subcutaneously inoculated into NSG mice, respectively. Three days later, the expression of GFP in a single tumor cell was analyzed by flow cytometry. *n* = 3. (F) Same as (E), but for H-stiff vector- or *ITGB8*-OE-B16 cells. (G) Ten M-stiff SGCTRL or *ITGB8*-SGs-MCF7 or B16 tumor cells embedded in 50 μl of fibrin gel (90 Pa) were inserted into the subcutaneous site of C57BL/6 or NSG mice (*n* = 20 for B16 cells or *n* = 16 for MCF-7 cells). The tumor formation was recorded. In (B) to (F), *n* = 3 biological independent experiments. ***P < 0.001, by 2-tailed Student’s *t* test (C and D) or one-way ANOVA Bonferroni’s test (B, E, and F). *NS*, no significant difference. The data represent mean ± SD.

Next, we investigated how the above mechanical cue was transduced to induce stiff tumor cell dedifferentiation. Given that β integrin mediates mechanotransduction [25,26], we determined the expression of all β subunits from β1 to β8, and found that β8 was markedly upregulated in melanoma and breast TRCs, compared to their differentiated counterparts (Fig. 2A and Fig. S2A and B). Notably, unlike other members, β8 is unique, because (a) it only binds αv to form a heterodimer [27], and (b) its cytoplasmic domain is unlikely to augment cell spreading and adhesion for cell differentiation [28]. We therefore used CRISPR/cas9 to knock out β8 and seeded β8^−/−^ stiff cells into 90-Pa fibrin gels. We found that β8-deficient cells did not form colonies (Fig. 2B and Fig. S2C to F). Using *ITGB8* promoter-GFP-expressing MCF-7 cells, we also observed that the expression of β8 was only upregulated in M-stiff cells but not in H-stiff tumor cells cultured in soft fibrin gels (Fig. 2C). Intriguingly, forced β8 overexpression allowed H-stiff MCF-7 cells to grow colonies in soft fibrin gels (Fig. 2D and Fig. S2G), suggesting that β8 is crucial for tumor cell dedifferentiation in a soft microenvironment. Notably, β8 seemed not to affect the stiffness of tumor cells, as evidenced by either *ITGB8* knockout or overexpression (Fig. S2H). In addition, the use of the RGD peptide to block αvβ8 abrogated colony formation by M-stiff cells in 90-Pa fibrin gels (Fig. S2I). To validate this notion in vivo, we inoculated β8-deficient, *SOX2* promoter-GFP tumor cells (~800 Pa stiffness) into mice. In contrast to their β8^+/+^ counterparts, β8-deficient cells could not be converted into GFP^high^ tumor cells in mice (Fig. 2E). However, β8-overexpressing, *SOX2* promoter-GFP tumor cells (~1,100 Pa stiffness) could be converted to GFP^high^ tumor cells in mice (Fig. 2F). In addition, we subcutaneously inserted a gel containing 10 SGCTRL or β8-deficient tumor cells into wild-type C57BL/6 or NSG mice. Twelve weeks later, we found that all NSG mice (20/20) and more than 30% of wild-type mice, inserted with SGCTRL tumor cells, developed a tumor, while tumor formation in the β8-deficient group was greatly reduced (Fig. 2G and Fig. S2J). However, upon β8 overexpression, tumor formation was regained from the tumor cells (~1,100 Pa stiffness) at a proportion of 13/20 in NSG mice or 5/20 in WT mice (Fig. S2K). Similar results were obtained for MCF-7, 4T1, and MP-1 cells (Fig. 2G and Fig. S2J to L). Together, these results suggest that β8 integrin mediates mechanotransduction for tumor cell dedifferentiation.

### YAP inactivation is linked to integrin β8-mediated mechanotransduction

**Fig. 3.**
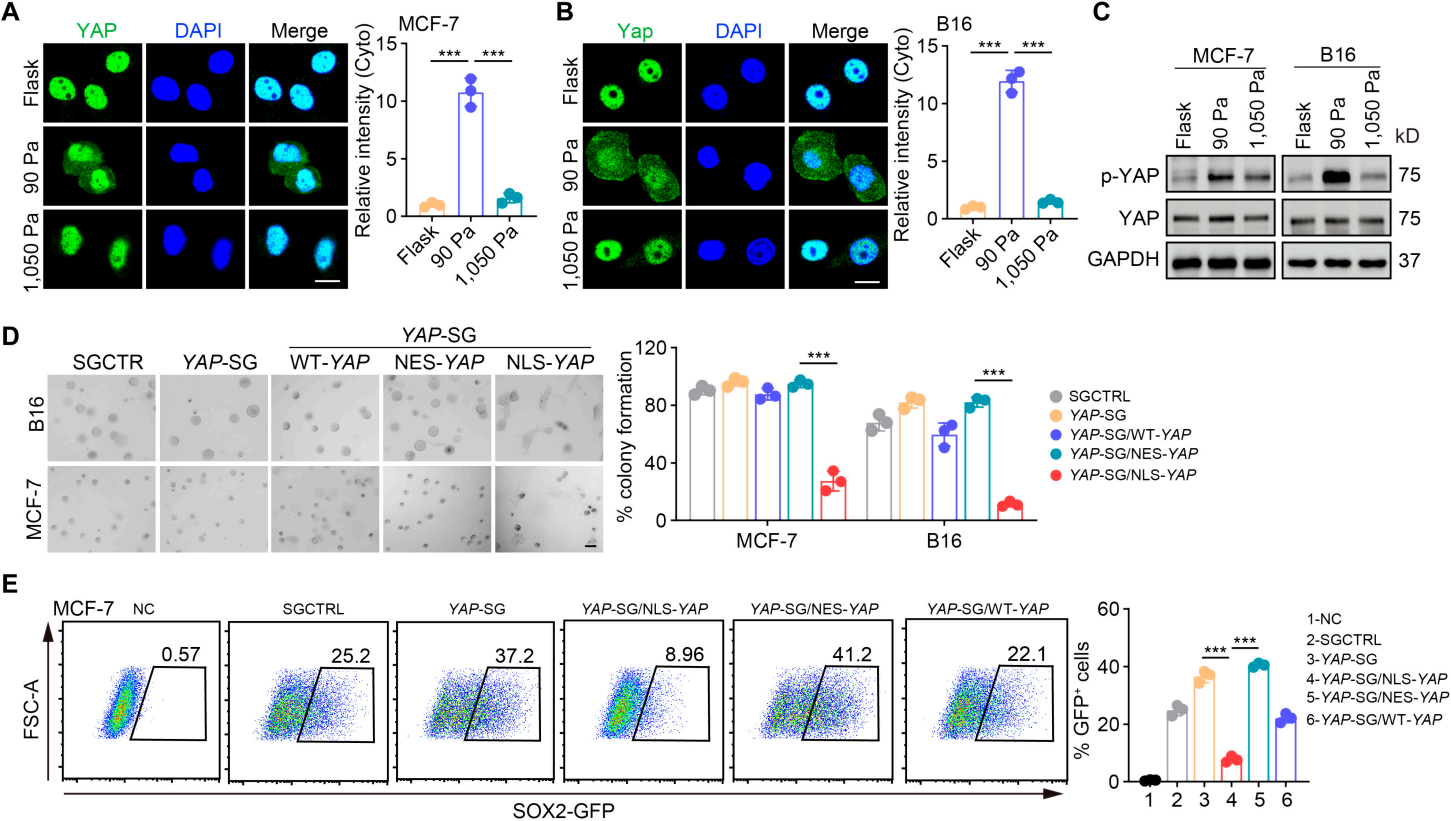
YAP inactivation is required for tumor cell dedifferentiation. (A and B) Immunostaining of YAP in MCF-7 (A) or B16 (B) cells cultured on a rigid plate or in the 90-Pa or 1,050-Pa 3D fibrin gel for 48 h. Scale bars, 10 μm. (C) The levels of p-YAP and total YAP protein in B16 or MCF-7 cells cultured in a flask or the 90-Pa or 1,050-Pa 3D fibrin gel for 48 h were analyzed by Western blot. (D) SGCTRL, *YAP*-SG, *YAP*-SG/NLS-*YAP*, *YAP*-SG/NES-*YAP*, or *YAP*-SG/WT-*YAP*-B16 or MCF-7 cells were seeded in the 90-Pa soft 3D fibrin gel for 4 days. The colonies were presented photographically (left) and the percentage of colony formation was calculated (right). Scale bar, 20 μm. (E) SGCTRL, *YAP*-SG, *YAP*-SG/NLS-*YAP*, *YAP*-SG/NES-*YAP*, or *YAP*-SG/WT-*YAP*-MCF-7 cells that were SOX2 promoter-GFP-expressing were seeded in the 90-Pa soft 3D fibrin gel for 48 h. The expression of GFP in a single MCF-7 cell was analyzed by flow cytometry. In (A), (B), (D), and (E), *n* = 3 biological independent experiments. ***P < 0.001, by one-way ANOVA Bonferroni’s test (A, B, D, and E). The data represent mean ± SD.

Next, we investigated the manner by which β8-mediated mechanotransduction induced tumor cell dedifferentiation. YAP, a crucial transcription activator, has been highlighted as a mechanical signal molecule that senses integrin-mediated mechanotransduction [29], and is also known as the Hippo signaling-phosphorylated substrate [30]. Intriguingly, we found that phosphorylated YAP was present in the cytoplasm of B16/MP-1 and 4T1/MCF-7 cells in 90-Pa soft fibrin gels, which, however, was translocated to the nucleus of the cells in the dephosphorylated form upon culturing in 1,050-Pa gels or rigid dishes (Fig. 3A to C and Fig. S3A to D). We therefore knocked out β8 and seeded M-stiff β8^−/−^ tumor cells into 90-Pa fibrin gels. As expected, YAP phosphorylation was abrogated by β8 deficiency (Fig. S3E), suggesting that YAP is regulated by β8-mediated mechanotransduction. In addition, knocking out both αv (partner of β8) and β8 further decreased the levels of p-YAP (Fig. S3F and G). To determine the role of YAP in tumor cell dedifferentiation, we constructed nuclear location signal (NLS)- and nuclear export signal (NES)-tagged YAP-expressing cells and found that NLS-YAP abrogated the above tumor cell dedifferentiation, while exporting YAP from the nucleus (NES-YAP) promoted dedifferentiation, as evidenced by colony formation and *SOX2* expression (Fig. 3D and E and Fig. S3H). Together, these results suggest that β8-mediated mechanotransduction inactivates YAP for dedifferentiation of tumor cells.

### YAP inactivation relies on RhoA inactivation

**Fig. 4.**
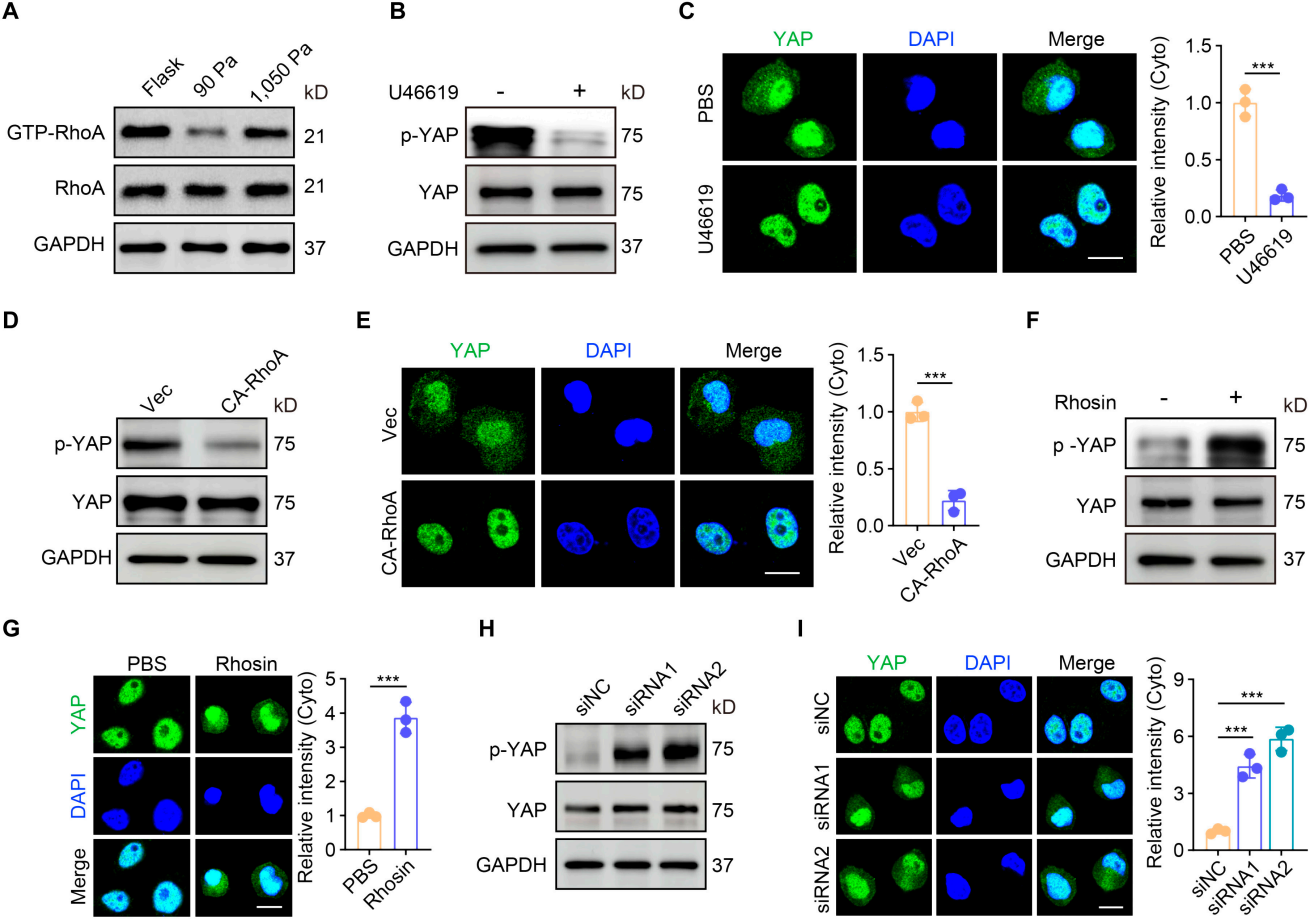
Integrin β8-mediated mechanotransduction inactivates YAP through regulating RhoA activity. (A) The levels of GTP-RhoA or total RhoA protein in MCF-7 cells cultured on a rigid plate or in the 90-Pa or 1,050-Pa 3D fibrin gel for 48 h were analyzed by Western blot. (B) The levels of p-YAP or total YAP protein in MCF-7 cells treated with U46619 (1 μM) when cultured in the 90-Pa soft 3D fibrin gel for 48 h were analyzed by Western blot. (C) Immunostaining of YAP in MCF-7 cells treated with U46619 (1 μM) when cultured in the 90-Pa soft 3D fibrin gel for 48 h. Scale bar, 10 μm. (D) The levels of p-YAP or total YAP protein in MCF-7 cells that were transfected with constitutively active RhoA (Q63L) (CA-RhoA) or vector cultured in the 90-Pa soft fibrin gels for 48 h were analyzed by Western blot. (E) Immunostaining of YAP in MCF-7 cells that were transfected with CA-RhoA or vector cultured in the 90-Pa soft 3D fibrin gel for 48 h. Scale bar, 10 μm. (F) Same as (B), except tumor cells were treated with Rhosin (10 μM). (G) Same as (C), except tumor cells were treated with Rhosin (10 μM) and cultured in the 1,050-Pa soft 3D fibrin gel for 48 h. Scale bar, 10 μm. (H) The levels of total YAP1 or p-YAP in MCF-7 cells that were transfected with RhoA siRNA or siNC cultured in the 1,050-Pa 3D fibrin gel for 48 h were analyzed by Western blot. (I) Immunostaining of YAP in MCF-7 cells that were transfected with RhoA siRNAs or siNC cultured in the 1,050-Pa 3D fibrin gel for 48 h. Scale bar, 10 μm. In (C), (E), (G), and (I), *n* = 3 biological independent experiments. ***P < 0.001, by 2-tailed Student’s *t* test (C, E, and G) or one-way ANOVA Bonferroni’s test (I). The data represent mean ± SD.

Next, we investigated the manner by which YAP was inactivated by β8-mediated mechanotransduction. The Hippo pathway uses large tumor suppressor homologs 1 and 2 (LATS1/2) to directly phosphorylate YAP [31]. However, knockout of LATS (LATS1/2-dKO) had a minor effect on the cytoplasmic location of YAP in these cells (Fig. S4A and B). In addition to the Hippo pathway, recent studies have revealed RhoA as an important activator of YAP/TAZ3 [30]. Coincidentally, RhoA activity may be inhibited by integrin engagement [32]. We thus hypothesized that RhoA inactivation mediates YAP phosphorylation. As expected, a low and a high RhoA activity of the MCF-7 or B16 cells were observed in soft 90-Pa fibrin gels and plastic plate, respectively (Fig. 4A and Fig. S4C). Increasing RhoA activity using its agonist U46619 blocked YAP phosphorylation in soft gels (Fig. 4B and C and Fig. S4D and E). By transfecting active RhoA (Q63L) (CA-RhoA) into tumor cells, we found that YAP phosphorylation was inhibited in soft fibrin gels (Fig. 4D and E and Fig. S4F and G). In addition, using a RhoA inhibitor Rhosin or knockdown of RhoA by siRNA resulted in increased YAP phosphorylation in MCF-7 or B16 cells seeded in the 1,050-Pa gels (Fig. 4F to I and Fig. S4H to M). Together, these results suggest that β8-mediated mechanotransduction inactivates YAP by regulating RhoA activity.

### RhoGDI1 is recruited to integrin β8 for RhoA inactivation

**Fig. 5.**
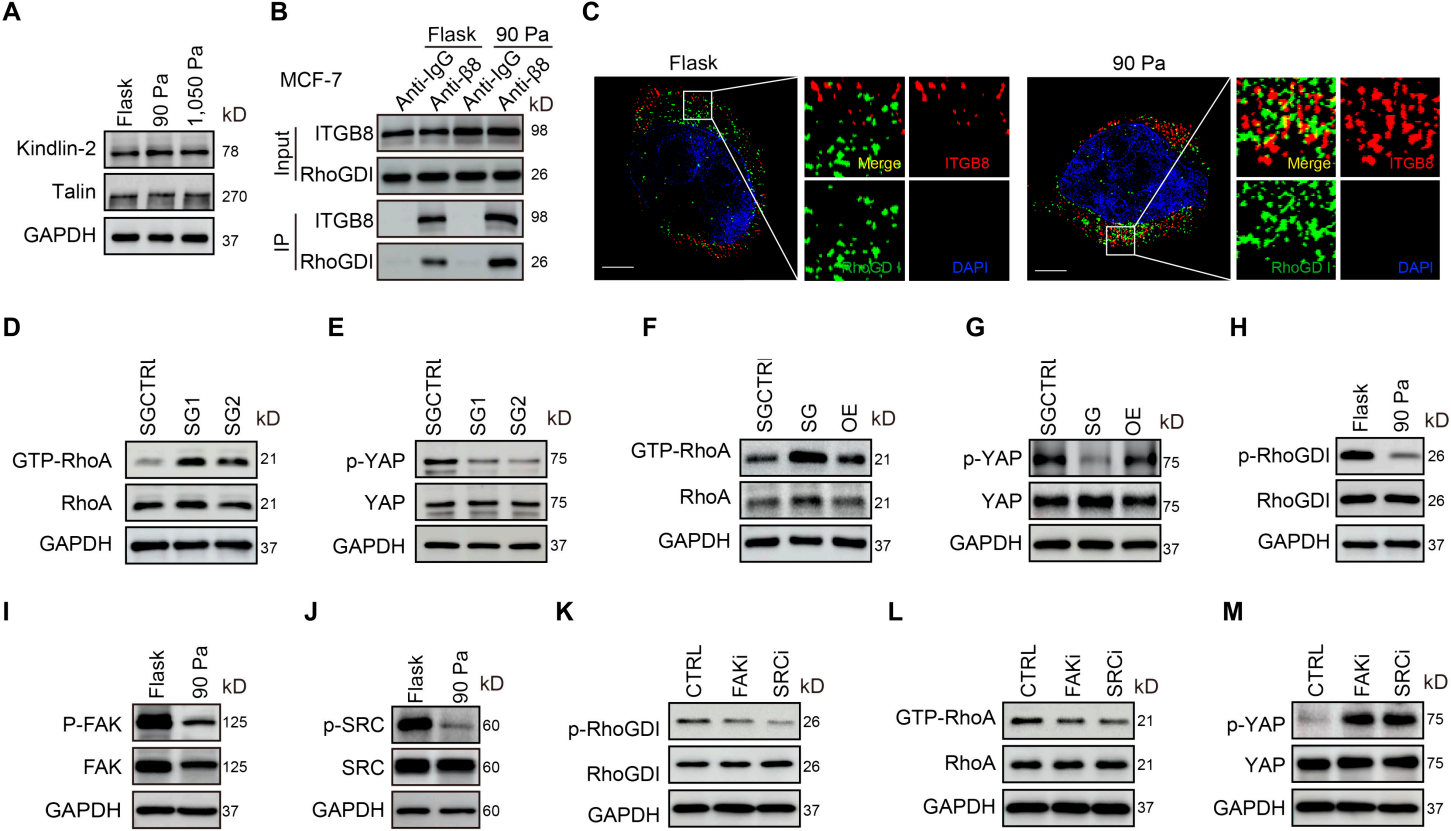
RhoGDI recruited by integrin β8 mediates RhoA inactivation in a soft mechanical microenvironment. (A) The levels of kindlin 2 and talin in MCF-7 cells cultured in a flask or the 90-Pa or 1,050-Pa 3D fibrin gel for 48 h were analyzed by Western blot. (B) MCF-7 cells were cultured in a flask or the 90-Pa 3D fibrin gel for 48 h. Cell lysates were collected for immunoprecipitation with anti-ITGB8 or anti-IgG antibody. The expression of RhoGDI and ITGB8 was analyzed by Western blot. (C) The MCF-7 cells cultured in a flask or the 90-Pa fibrin gel were stained with anti-RhoGDI and ITGB8 antibodies and observed under ultrahigh super-resolution microscopy. Scale bar, 5 μm. (D and E) The levels of GTP-RhoA, total RhoA (D), p-YAP, or total YAP (E) in SGCTRL or *RhoGDI*-SGs-MCF-7 tumor cells were analyzed by Western blot. (F and G) Same as (D) and (E), except for the SGCTRL, *RhoGDI*-SG, or *RhoGDI*-SG*/RhoGDI*-OE MCF-7 tumor cells. (H) The levels of p-RhoGDI or total RhoGDI in MCF-7 cells cultured in a flask or the 90-Pa 3D fibrin gel for 48 h were analyzed by Western blot. (I and J) The levels of p-FAK, FAK (I), p-SRC, or SRC (J) in MCF-7 cells cultured in a flask or the 90-Pa 3D fibrin gel for 48 h were analyzed by Western blot. (K to M) The levels of p-RhoGDI (K), RhoGDI (K), GTP-RhoA (L), RhoA (L), p-YAP (M), or YAP (M) in MCF-7 cells treated with PBS, SRC inhibitor (Dasatinib, 5 μM), or FAK inhibitor (GSK2256098, 5 μM) for 48 h were analyzed by Western blot.

Next, we investigated how β8-transduced mechanical signal inactivated RhoA. Although integrin β commonly contains NPXY motifs, the β8 cytoplasmic domain is divergent and lacks these motifs [27,33]. Kindlin 2, an NPXY motif-interacting cytoskeletal protein, promotes YAP activation [34]. We found that kindlin 2 knockdown did not affect the dedifferentiation of MCF-7 tumor cells in 90-Pa 3D fibrin gels (Fig. S5A to D). A similar result was obtained from the knockdown of talin, another NPXY motif-interacting protein (Fig. S5E to H). In addition, we found that talin and kindlin 2 were equally expressed among tumor cells cultured in a flask or in 90-Pa and 1,050-Pa fibrin gels (Fig. 5A and Fig. S5I). Thus, β8-mediated mechanotransduction does not seem to use talin or kindlin for RhoA regulation. Rho GDP dissociation inhibitor (RhoGDI) is an important cytoplasmic protein that inactivates RhoGTPases [35]. Upon RhoGDI binding, Rho GTPases are present in the cytoplasm; however, phosphorylation of RhoGDI by Sac or other kinases releases RhoGDI from RhoGTPases and allows RhoGTPase to be inserted into the plasma membrane for functional exertion. Notably, Rho GDP dissociation inhibitor 1 (RhoGDI1) has been reported to interact with the cytoplasmic tail of integrin β8 [36,37], providing a possibility that RhoGDI1was mobilized to inactivate RhoA. Performing the co-immunoprecipitation, we found that RhoGDI1 was bound with β8 (Fig. 5B), and ultrahigh super-resolution microscopy showed that RhoGDI1 colocalized with β8 on the plasma membrane (Fig. 5C). Knocking out RhoGDI1 enhanced both RhoA activity and YAP dephosphorylation; in contrast, overexpressing RhoGDI1 led to inhibition of RhoA and YAP activities (Fig. 5D to G and Fig. S5J to O). Src phosphorylates RhoGDI1 on Y156 [38]. We found that this phosphorylation was reduced in the above soft gel-cultured tumor cells (Fig. 5H and Fig. S5P). Focal adhesion kinase (FAK) positively regulates Src phosphorylation and RhoA activity [39]. FAK expression and phosphorylation were reduced in 90-Pa fibrin gels (Fig. 5I and Fig. S5Q), consistent with a previous report [40]. In line with this result, Src phosphorylation was also reduced (Fig. 5J and Fig. S5R). Treatment with either SRC or FAK inhibitor increased RhoGDI1 activity, but decreased RhoA activity and YAP dephosphorylation (Fig. 5K to M and Fig. S5S to U). In addition to RhoGDI1, protein tyrosine phosphatase non-receptor type 12 (PTP-PEST) is a tyrosine phosphatase, which can also bind to integrin β8, providing a possibility of dephosphorylating RhoGDI1. However, knockdown of PTP-PEST did not alter the dephosphorylation of RhoGDI1 (Fig. S5V to X). Meanwhile, PTP-PEST was lowly expressed in tumor cells cultured in a flask or soft fibrin gels (Fig. S5Y). Together, these results suggest that integrin β8 recruits RhoGDI1 to inactivate RhoA under the soft matrix condition.

### YAP inactivation relieves MAFG for stemness gene transactivation

**Fig. 6.**
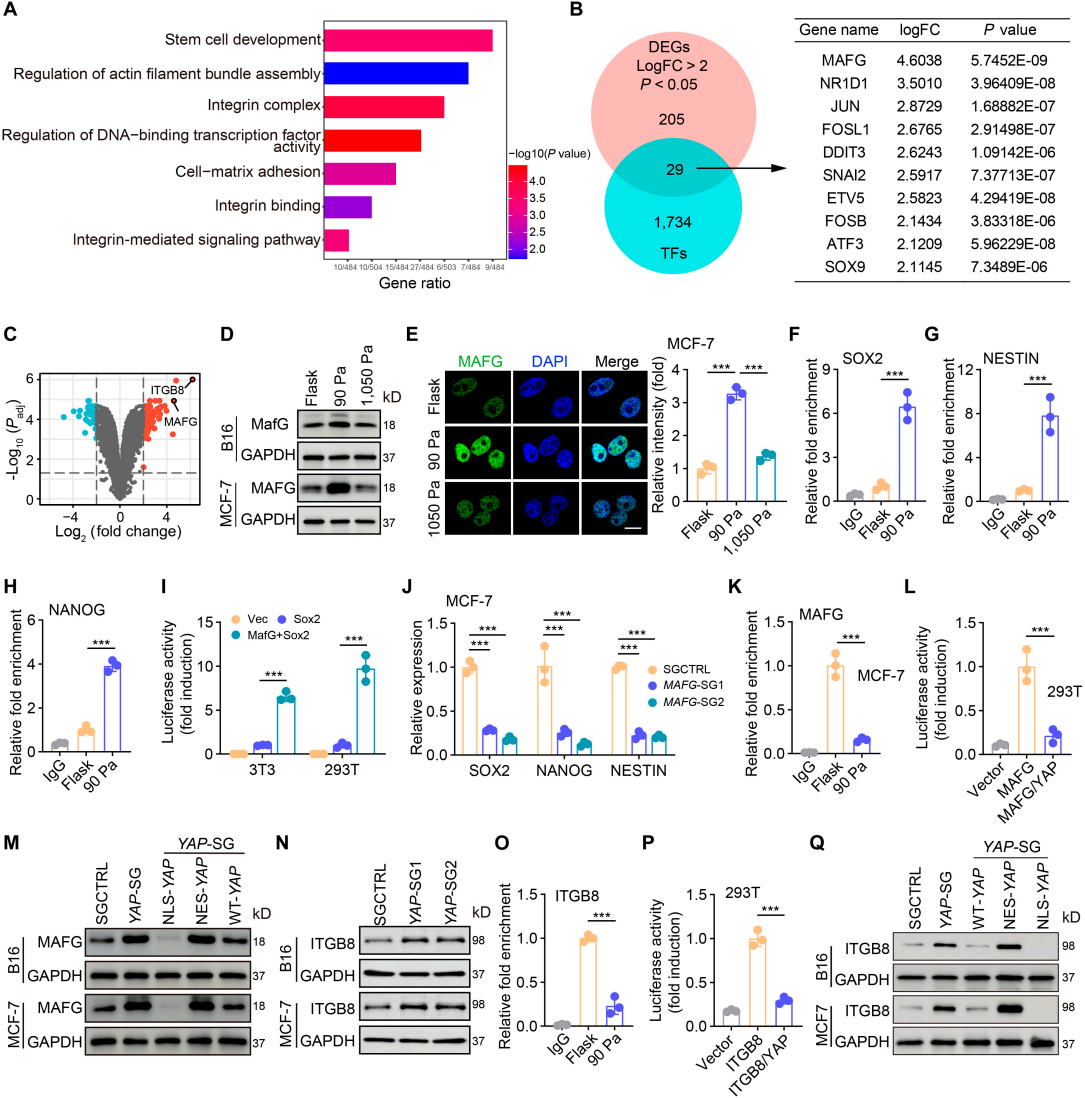
MAFG that released the transcriptional suppression from YAP promotes the stemness gene expression for tumor cell dedifferentiation. (A) MCF-7 cells were cultured on a rigid plate or in the 90-Pa fibrin gel for 48 h and then collected for RNA-seq. GO pathway enrichment analysis (*P* < 0.05) of upregulated differentially expressed genes (DEGs) in dedifferentiated MCF-7 cells. (B) Venn diagram of intersection of DEGs and transcription factor proteins in soft and stiff MCF-7 cells. The top 10 intersected genes were listed. The cutoff criteria of upregulated DEGs were log2 fold change > 2 and an adjusted *P* < 0.05. (C) Volcano plot of RNA-seq DEGs. The red dots and blue dots represent upregulated and downregulated DEGs based on a log-fold change of >1 and adjusted *P* < 0.05. *ITGB8* and *MAFG* were shown as red dots. (D) The level of MAFG in B16 or MCF-7 cells cultured on a rigid plate or in the 90-Pa or 1,050-Pa 3D fibrin gel for 48 h was analyzed by Western blot. (E) Immunostaining of MAFG in MCF-7 cells cultured on a rigid plate or in the 90-Pa or 1,050-Pa 3D fibrin gel for 48 h. Scale bar, 10 μm. (F to H) ChIP-qPCR analysis was performed with an antibody to MAFG and *SOX2* (F)*, NESTIN* (G), or *NANOG* (H)-promoter-specific primers in MCF-7 cells cultured on a rigid plate or in the 90-Pa 3D fibrin gel for 4 days. (I) The NIH3T3 or HEK293T cells were co-transfected with *SOX2* promoter-luciferase reporter PGL3 and pCMV-MAFG plasmid for 24 h followed by analysis of luciferase activity. (J) The expression of *SOX2*, *NESTIN*, or *NANOG* in SGCTRL or MAFG-SGs-MCF-7 cells cultured in the soft 3D fibrin gel for 48 h was analyzed by qPCR. (K) ChIP-qPCR analysis was performed with an antibody to YAP and *MAFG*-promoter-specific primers in MCF-7 cells cultured on a rigid plate or in the 90-Pa 3D fibrin gel for 4 days. (L) HEK293T cells were co-transfected with MAFG promoter-luciferase reporter PGL3 and pCMV-YAP plasmid for 24 h followed by analysis of luciferase activity. (M) The level of MAFG in SGCTRL, *YAP*-SG, *YAP*-SG/NLS-*YAP*, *YAP*-SG/NES-*YAP*, or *YAP*-SG/WT-*YAP*-B16 or MCF-7 cells cultured in the 90-Pa 3D fibrin gel was analyzed by Western blot. (N) The levels of ITGB8 in SGCTRL or YAP-SGs-B16 or MCF-7 cells cultured in the 90-Pa 3D fibrin gel were analyzed by Western blot. (O) Same as (K), except the ChIP-qPCR analysis was performed with *ITGB8*-promoter-specific primers. (P) Same as (L), except cells were transfected with ITGB8 promoter-luciferase reporter PGL3. (Q) The level of ITGB8 in SGCTRL, *YAP*-SG, *YAP*-SG/NLS-*YAP*, *YAP*-SG/NES-*YAP*, or *YAP*-SG/WT-*YAP*-B16 or MCF-7 cells cultured in the 90-Pa 3D fibrin gel was analyzed by Western blot. In (E) to (L), (O), and (P), *n* = 3 biological independent experiments. ***P < 0.001, by one-way ANOVA Bonferroni’s test (E to L, O, and P). The data represent mean ± SD.

Next, we explored the manner by which YAP phosphorylation resulted in tumor cell dedifferentiation. One possible mechanism was the direct regulation of stemness genes by YAP. However, chromatin immunoprecipitation-quantitative polymerase chain reaction (ChIP-qPCR) did not show the binding of YAP to the promoter regions of *Nanog*, *Sox2*, and *Nestin* (Fig. S6A). RNA sequencing of soft (90 Pa fibrin gel) and stiff (rigid plate) MCF-7 cells revealed that the upregulated differentially expressed genes (DEGs) in soft cells were enriched in transcription-related pathways (Fig. 6A and Table S1). We thus used the upregulated DEGs to intersect with the Transcription Factor Protein Database [41]. Among the screened transcription factors, MAFG, a small Maf protein that belongs to the basic leucine zipper family of transcription factors [42–44], drew our attention and had the highest score (Fig. 6B and C). Both real-time PCR and Western blot showed that MAFG was upregulated in soft cells, compared to stiff cells (Fig. 6D and Fig. S6B). Intriguingly, we found that the MAFG was present in the nucleus of the cells and was upregulated in soft fibrin gels (Fig. 6E and Fig. S6C), indicating a possibility that MAFG regulates dedifferentiation. The ChIP-qPCR showed that MAFG bound to the promoter region of stemness genes *NANOG*, *SOX2*, and *NESTIN* (Fig. 6F to H and Fig. S6D to F). Luciferase assay demonstrated that MAFG transactivated these genes (Fig. 6I and Fig. S6G to J). Knocking out or overexpressing MAFG resulted in the downregulation or upregulation of the stemness genes in 90-Pa fibrin gel-cultured tumor cells (Fig. 6J and Fig. S6K to N), suggesting that MAFG mediates the above tumor cell dedifferentiation. In addition, MAFG overexpression upregulated stemness genes in the rigid-plate-cultured tumor cells (Fig. S6O and P). Then, we determined whether YAP transcriptionally repressed MAFG expression. ChIP-PCR and luciferase results showed that YAP bound to the MAFG promoter and suppressed MAFG expression (Fig. 6K and L). In addition, we found that NLS-YAP or NES-YAP markedly downregulated or upregulated MAFG expression in soft fibrin gel-cultured tumor cells (Fig. 6M). In line with this, YAP knockout upregulated the expression of *SOX2, NANOG*, and *NESTIN*, which, however, was abrogated by MAFG knockdown (Fig. S6Q and R). In addition to relieving the repression of MAFG, we found that YAP knockout also led to the upregulation of β8 expression (Fig. 6N). ChIP-PCR and luciferase assays confirmed that YAP bound to the β8 promoter and inhibited β8 expression (Fig. 6O and P). In addition, we found that NLS-YAP or NES-YAP downregulated or upregulated β8 expression (Fig. 6Q), and the knockout or overexpression of MAFG did not alter β8 expression (Fig. S6S). Therefore, it was YAP rather than MAFG that regulated β8 expression. Given that YAP performs its function by binding different partners, such as P73, TEAD, and RUNX2 [45], we further knocked down *P73*, *TEAD*, and *RUNX2* to determine the possible role in the regulation. We found that the knockdown of *RUNX2* but not *TEAD* or *P73* upregulated the expression of MAFG and stemness genes in stiff tumor cells (Fig. S7A to H). In line with this, the binding of YAP with RUNX2 was upregulated in the stiff tumor cells (Fig. S7I). Together, these results suggest that YAP phosphorylation relieves the repression of *MAFG* and *β8* genes, thus promoting stemness gene expression and forming a mechano-regulatory loop for tumor cell dedifferentiation.

### MAFG expression is a potential prognostic marker for cancer patients

**Fig. 7.**
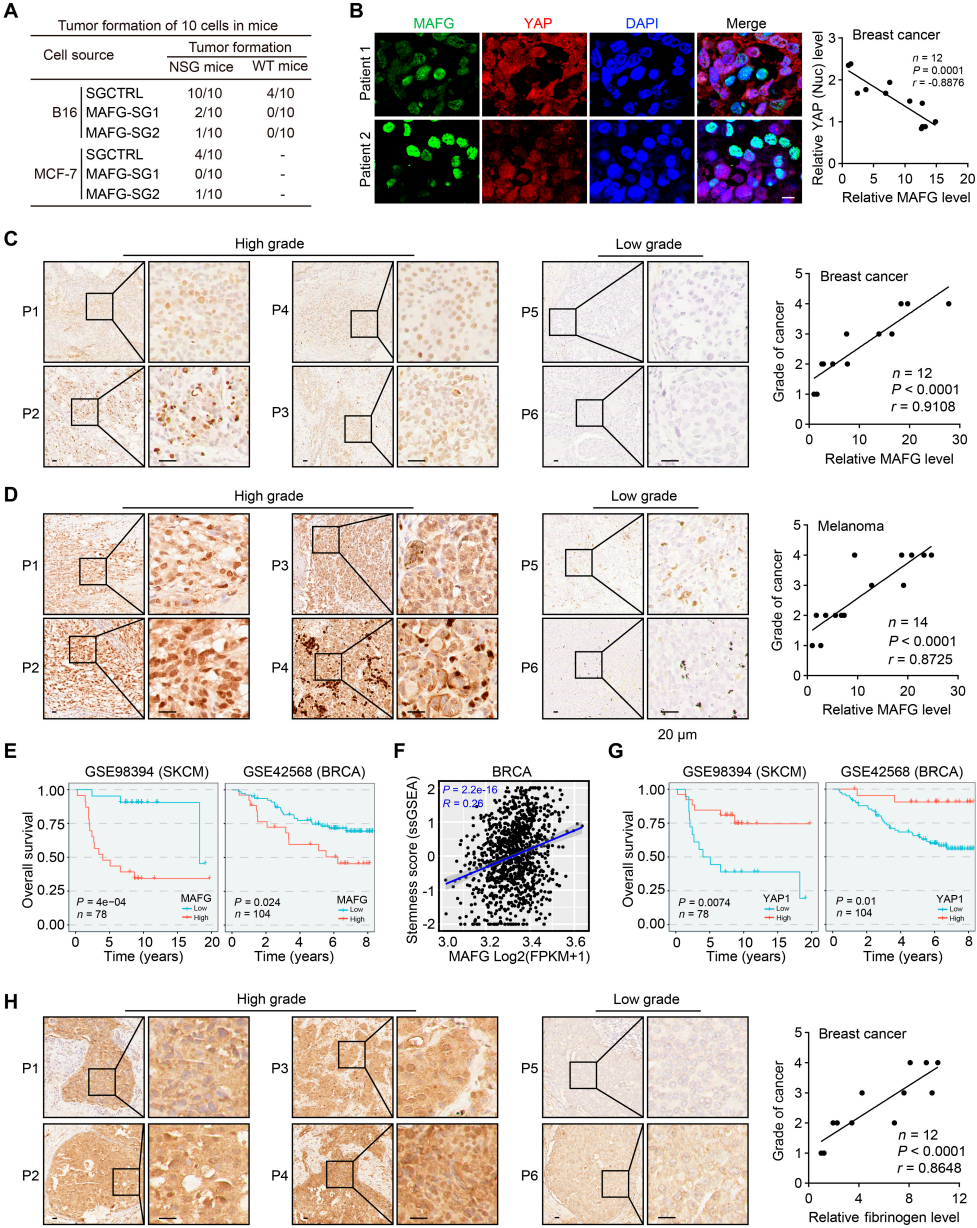
MAFG expression is a potential prognostic marker for cancer patients. (A) Ten M-stiff SGCTRL or *MAFG*-SGs-B16 or MCF-7 tumor cells embedded in 50 μl of fibrin gel (90 Pa) were inserted into the subcutaneous site of C57BL/6 or NSG mice (*n* = 10). The tumor formation was recorded. (B) The tissue sections from 12 breast cancer patients underwent immunofluorescence staining with anti-YAP1 and anti-MAFG antibody. The correlation between the expression of YAP1 and MAFG was shown (right). Scale bars, 10 μm. (C) The tissue sections from 4 breast cancer patients with high grade of malignancy (left) and 2 breast cancer patients with low grade of malignancy (middle) underwent immunohistochemical staining with anti-MAFG antibody. The correlation between the expression of MAFG from 12 patients with breast cancer and tumor pathological grade was shown (right). Scale bars, 50 μm. (D) The tissue sections from 4 melanoma patients with high grade of malignancy (left) and 2 melanoma patients with low grade of malignancy (middle) underwent immunohistochemical staining with anti-MAFG antibody. The correlation between the expression of MAFG from 14 patients with melanoma and tumor pathological grade was shown (right). Scale bars, 50 μm. (E) Overall survival compared with the MAFG expression in patients with SKCM (GSE98394, *n* = 78) and BRCA (GSE42568, *n* = 104). (F) TCGA database RNA-seq analysis of the correlation between the expression of *MAFG* and stemness score in people with BRCA (*n* = 1,097). (G) Overall survival compared with the YAP expression in patients with SKCM (GSE98394, *n* = 78) and BRCA (GSE42568, *n* = 104). (H) The tissue sections from 4 breast cancer patients with high grade of malignancy (left) and 2 breast cancer patients with low grade of malignancy (middle) underwent immunohistochemical staining with anti-fibrinogen antibody. The correlation between the expression of fibrinogen from 12 patients with breast cancer and tumor pathological grade was shown (right). Scale bars, 50 μm. **P < 0.01, ***P < 0.001, by Spearman’s correlation test (B to D, F, and H) or log-rank survival analysis (E and G). The data represent mean ± SD.

Identification of the regulation of tumor cell dedifferentiation by the β8-RhoGDI1-RhoA-YAP-MAFG mechanical signaling pathway prompted us to validate the potential translation of MAFG in cancer patients. When we subcutaneously inserted a small piece of the 90-Pa fibrin gel containing 10 B16 cells into wild-type C57BL/6 or NSG mice, we found that compared to SGCTRL B16 cells, MAFG knockout strikingly decreased the tumor formation of B16 cells in NSG mice (10/10 versus 2/10) and in wild-type mice (4/10 versus 0/10) (Fig. 7A). Similar results were obtained for MCF-7 cells in the NSG mice (Fig. 7A). Immunostaining of primary human breast cancer samples (*n* = 12) revealed that MAFG was negatively correlated with nuclear YAP (Fig. 7B) and positively correlated with the tumor pathological grade (Fig. 7C). Immunostaining of human melanoma samples (*n* = 14) also showed a positive correlation between MAFG and tumor pathological grade (Fig. 7D). Using The Cancer Genome Atlas (TCGA) and Gene Expression Omnibus (GEO) databases of RNA sequencing (RNA-seq) data, we found that MAFG expression was negatively correlated with BRCA and SKCM (Fig. 7E and Fig. S8A) patient survival, which also had a positive correlation with the stemness score of the tumors (Fig. 7F). In addition, MAFG expression also negatively correlated with the survival of patients with liver hepatocellular carcinoma (*n* = 374) (Fig. S8B), uterine corpus endometrial carcinoma (*n* = 552) (Fig. S8C), lung squamous cell carcinoma (*n* = 502) (Fig. S8D), bladder urothelial carcinoma (*n* = 414) (Fig. S8E), and esophageal carcinoma (*n* = 162) (Fig. S8F). In addition, by analyzing previously reported data (GSE98394 and GSE9893), we found that the overall survival of patients with BRCA (*n* = 104) or SKCM (*n* = 78) was positively correlated with YAP expression (Fig. 7G). Considering the induction of MAFG expression in the fibrin-based mechanical environment, here we also analyzed fibrinogen levels in clinical breast tumor samples (*n* = 12). We found that fibrinogen levels were positively correlated with MAFG expression and the tumor pathological grade (Fig. 7H and Fig. S8G to J). These results suggest that MAFG expression may be a potential prognostic marker for tumor patients.

## Discussion

Cells use contractile filaments to sense the mechanical force of the surrounding microenvironment and respond accordingly [21,46]. Collagen and fibrin, 2 extracellular matrix proteins, bind to integrins, causing mechanotransduction along clumped integrins to focal adhesions [3]. Thus, external mechanical signals converge to focal adhesions and can be converted into biochemical signals. In line with this principle, in this study, we provide evidence that integrin β8 induces tumor cell dedifferentiation, one of the core biological events in malignancy, by transducing a soft mechanical force.

One substantial finding of this study is that tumor cells with moderately high rather than the highest stiffness have the ability to dedifferentiate. Stiffness is an intrinsic cell property. We previously showed that stem-cell-like tumor cells are very soft (stiffness < 400 Pa) and differentiated tumor cells are stiff (stiffness > 700 Pa) [11]. However, stiffness may reflect continuous differentiation, and the highest stiffness indicates terminal differentiation. Cellular stiffness is decided by tensile stress, which is generated from actin microfilament structures [47,48]. The tensile tension in actin filaments increases with the activation of myosin-based contraction, stiffening the F-actin lattice in the process [21,49]. Thus, cell stiffness is the collective result of actin polymerization and myosin II-mediated contractile activation. Since microfilament-mediated contraction is sensed by the extracellular matrix (ECM) via integrins, cell stiffness should be matched with ECM stiffness. Thus, terminally differentiated tumor cells use more microfilaments to adapt to the highly stiff mechanical microenvironment, and the corresponding mechanical signal transduction is converted to proper chemical signals in the cytosol for biological behaviors of stiff tumor cells. However, if highly stiff tumor cells (well-differentiated) are within a soft mechanical microenvironment, the unsuitability of the mechanical cue is too large to allow cell dedifferentiation. In contrast, moderately stiff tumor cells may adapt to such soft microenvironments and be induced to dedifferentiate.

**Fig. 8.**
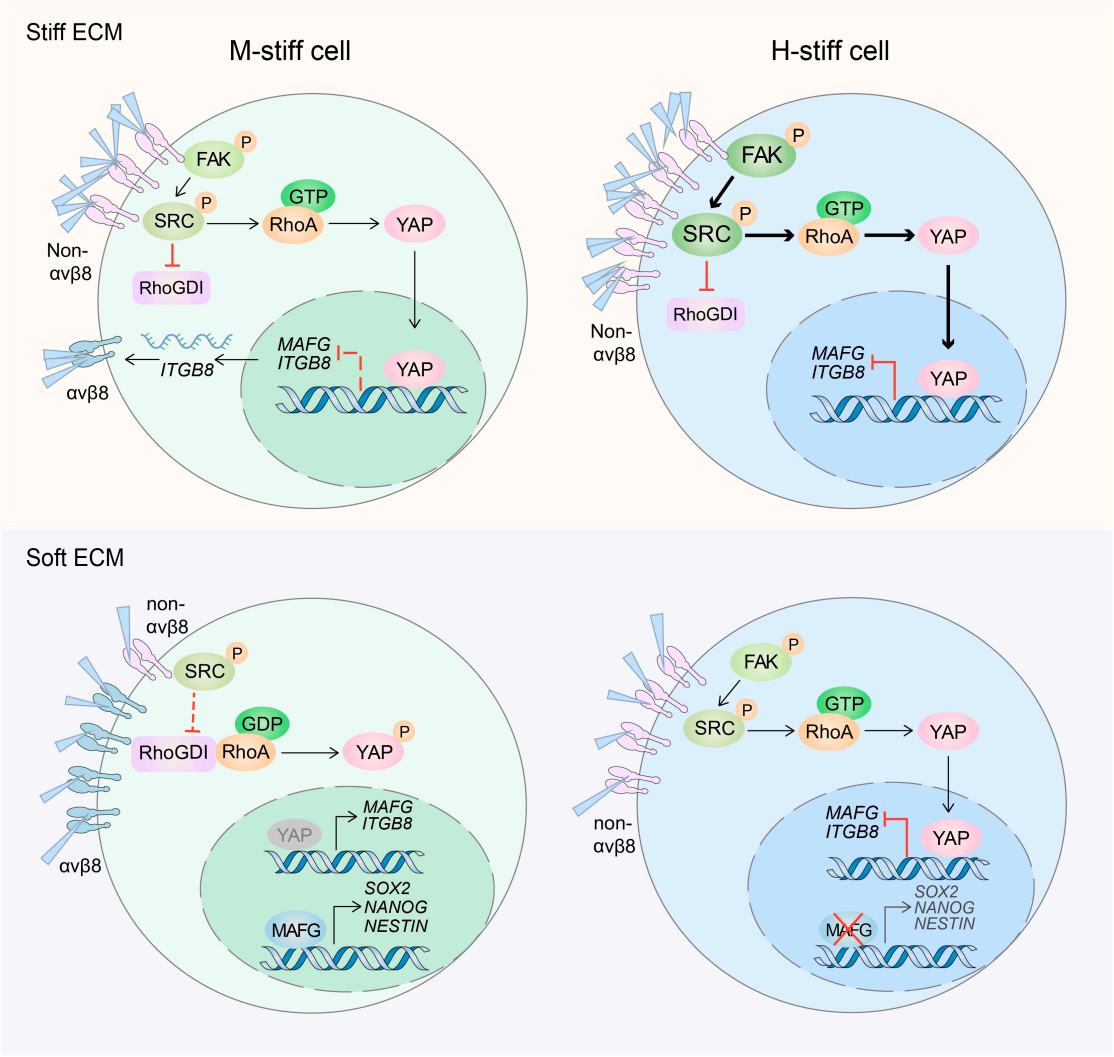
Schematic diagram of the ITGB8-YAP-MAFG pathway regulating the dedifferentiation in soft mechanical microenvironments. H-stiff tumor cells have high Src and FAK activities that result in strong YAP nuclear localization, where YAP binds to the MAFG gene promoter and heavily represses MAFG expression; M-stiff tumor cells have moderate Src and FAK activities and generate moderately strong YAP nuclear location to inhibit MAFG and β8 expression; in a soft mechanical environment such as 90-Pa fibrin gels, the low mechanotransduction may further weaken Src and FAK activities in M-stiff cells and relieve more repression of MAFG and β8; and as a positive feedback, the upregulated β8, upon sensing the mechanical force, binds more RhoGDI1 to inactivate YAP and promote tumor cell dedifferentiation.

Another important finding of this study was that YAP inactivation mediates M-stiff tumor cell differentiation in the soft matrix. YAP has been demonstrated to be a mechanosensor and mechanotransducer for regulating tumor growth [50,51]. YAP is localized in the cytoplasm of cells grown in a soft matrix, whereas it is localized in the nucleus of cells grown in a stiff matrix [52], suggesting that the subcellular localization of YAP is strictly controlled by matrix stiffness. In line with this notion, our present study also showed that YAP is present in the cytosol in an inactive form under soft matrix conditions. Notwithstanding this consistency, the role of YAP in tumor cell stemness is controversial. Although studies have reported that YAP activation is required for the development of tumor cell stemness [53,54], reports have also shown that YAP inactivation promotes stem-cell-like tumor cell growth by downregulating Nupr1 and even YAP exerts anticancer activity [55,56]. In addition, low YAP1 levels prevent hematologic malignancies from inducing apoptosis and negatively regulate mesenchymal stem cell differentiation [57,58]. In the present study, we demonstrate that YAP inactivation is required for tumor cell dedifferentiation and stemness. We provide a clear molecular basis for the following: (a) integrin β8 senses a soft mechanical signal, thus recruiting RhoGDI1 for RhoA inactivation and YAP phosphorylation; (b) YAP inactivation relieves the repression of MAFG, thus allowing MAFG to transactivate stemness gene expression; and (c) YAP inactivation also relieves the repression of β8, leading to the upregulation of β8 expression to form a mechano-regulation loop. We also provided evidence that MAFG transactivates the expression of stemness genes, including *SOX2*, *NANOG*, and *NESTIN*. Based on this mechanistic elucidation, we propose a moderate stiffness theory for tumor cell dedifferentiation. As shown in Fig. 8, (a) H-stiff tumor cells have high Src and FAK activities that result in strong YAP nuclear localization, where YAP binds to the promoter of MAFG gene and heavily represses MAFG expression; (b) M-stiff tumor cells have moderate Src and FAK activities and generate moderately strong YAP nuclear location to inhibit MAFG and β8 expression; (c) in a soft mechanical environment such as 90-Pa fibrin gels, the low mechanotransduction may further weaken Src and FAK activities in M-stiff cells and relieve more repression of MAFG and β8; and (d) as a positive feedback, the upregulated β8, upon sensing the mechanical force, binds more RhoGDI1 to inactivate YAP and promote tumor cell dedifferentiation. However, the question remains as to why H-stiff tumor cells are unable to dedifferentiate in the soft gel, since YAP activity is also decreased in the soft mechanical environment. Here, we further propose that distinct epigenetic programs are involved in dedifferentiation. That is, stronger epigenetic repression is present in H-stiff cells, which forms another brake besides YAP, thus preventing H-stiff cells from expressing MAFG and β8 in a soft gel. Nevertheless, this proposal should be supported by substantial experimental results obtained from future studies.

Our findings have implications for the development of novel anticancer therapeutics. Various treatment approaches are able to effectively eliminate tumor cells; however, they generate disappointing outcomes due to remnant treatment-resistant CSCs [59]. Thus, targeting stem-like tumor cells is currently the intent to deprive tumors of their ability to regenerate and thrive. However, these ideas face the challenge of previous studies and our findings that some differentiated tumor cells have the ability to dedifferentiate into CSCs [2]. Thus, even if targeting CSCs is valid, the dedifferentiation event may replenish the stem-like tumor cell population. Thus, targeting CSC populations might be unlikely to yield durable clinical responses. Treatment-resistant CSCs are considered to be selected from bulk tumor cells in which the treatment-sensitive tumor cells are eliminated. However, our findings suggest that treatment may facilitate tumor cell dedifferentiation. Tumor tissues are generally hard, and stiff matrices are unsuitable for tumor cell dedifferentiation. However, treatment-induced inflammation and enzymes may result in matrix degradation, thus generating a soft mechanical signaling that favors tumor cell dedifferentiation [56,60]. Based on our findings, targeting the integrin β8–MAFG axis might dampen treatment-induced tumor cell dedifferentiation.

In summary, this study’s data unequivocally demonstrate that partially differentiated tumor cells, by virtue of their mechanical features, respond to soft matrices and thereby dedifferentiate into highly tumorigenic cells through an integrin β8-mechanotransduced signaling pathway. Our research identifies possible targets for upcoming cancer therapies aimed at eradicating stem-like tumor cells.

## Materials and Methods

### Animals and cell lines

The Charles River Laboratories or the Center for Experimental Animal Research (CEAR) of CAMS provided the 6-week-old female C57BL/6, BALB/c, and NOD/SCID IL-2R-null mice (NSG). These animals were kept in a pathogen-free environment at CEAR. The Animal Care and Use Committee at CAMS (ACUC-A02-2023-016) gave its clearance to all experiments using mice. The National Biomedical Cell-Line Resource (BMCR) of China provided the human MCF-7 breast cancer cell line, the 4T1 breast cancer cell line, and the murine B16 melanoma cell line. RPMI1640 medium (Gibco, USA) was used to cultivate primary human MP-1 melanoma tumor cells that were obtained from human melanoma tissue. The 4T1 cell line was cultivated in RPMI1640 media, whereas B16 and MCF-7 cells were maintained in Dulbecco’s Modified Eagle Medium (Gibco, USA). Ten percent fetal bovine serum and 2 mM L-glutamine were added to all media (Gibco, USA). All cells were grown in an incubator with 5% CO_2_ at 37 °C. Before the investigation, cells were examined for the presence of mycoplasma, checked for cross-species contamination, and verified using isoenzyme and short tandem repeat analysis in BMCR.

### Plasmids and reagents

From Addgene (MA, USA), PX459 and pEF1a-IRES-Puro were acquired. *ITGB8*, *YAP1*, *MAFG*, and *RhoGDI* cDNAs came from Sino-Biological. DNA sequencing was used to validate every plasmid. These cDNAs were added to the lentiviral vector plasmid pLV-EF1-IRES-Puro (Addgene, 85132) with a carboxy-terminal 3Flag tag for transient expression in 293T cells to produce the lentivirus containing the target genes, either with or without the nuclear localization signal (NLS) (PKKKRKV) or the NES (LALKLAGLDI). With *YAP1*-SGs B16 and MCF-7, the lentiviruses carrying NLS, NES, or WT-*YAP1* were transduced. Selleck (TX, USA) produces the FAK inhibitor GSK2256098 as well as the SRC inhibitor Dasatinib.

### Human samples

The National Cancer Center/Cancer Hospital provided the paraffin-embedded tumor tissues of melanoma and breast cancer patients. Ethical permission was granted by the Medical Ethics Committee of Peking Union Medical College (SB2023001). Tables S2 and S3 included a list of the patients’ clinical data.

### Tumor cells cultured on rigid plastic plate or in 3D fibrin gels

Tumor cells were maintained in a hard plate with full culture media for traditional 2D cell culture. TRC culture was performed according to our previously published protocol [12]. The 3D fibrin gels were treated with dispase II (Roche) for 10 min at 37 °C to harvest the TRCs.

### Analysis of cellular stiffness and harvest of single tumor cell by FluidFM

A FluidFM BOT system (Cytosurge AG, Switzerland) carried out FluidFM, a method that not only can determine the stiffness of tumor cells but also can harvest a single tumor cell. In a nutshell, the FluidFM BOT’s micropipette probe was chosen because of its 4 μm and 0.2 N/m spring constant. The probe was filled up with 2 μl of 2.5% trypsin solution, coated by PAcrAm-g-(PMOXA, NH_2_, Si) medium for 20 min. After detection of the Young’s modulus, 2.5% trypsin solution was loaded in the channel of FluidFM and locally delivered to individual tumor cells, which activated the selective detachment of the targeted cell. The detached cell was then lifted up and collected for further manipulations.

### Western blotting

Using RIPA (radioimmunoprecipitation assay) lysis buffer, cells were lysed. A BCA (bicinchoninic acid assay) kit from Beyotime in China was used to determine the protein concentrations. The protein was then transferred to nitrocellulose after being processed on an SDS-PAGE (SDS–polyacrylamide gel electrophoresis) gel. After being blocked in 5% bovine serum albumin, the nitrocellulose membranes were overnight probed with antibodies: anti-GAPDH (Cell Signaling, Cat. no. 5174; 1:1,000), anti-ITGB8 (GeneTex, Cat. no. GTX64493; 1:1,000), anti-p-YAP (Cell Signaling, Cat. no. 13008; 1:1,000), anti-YAP (Cell Signaling, Cat. no. 14074; 1:1,000), anti-RhoA (Cell Signaling, Cat. no. 2117; 1:1,000), anti-Kindlin 2 (Abcam, Cat. no. ab194967; 1:1,000), anti-Talin 1 (Cell Signaling, Cat. no. 4021; 1:1,000), anti-RhoGDI (Cell Signaling, 2564; 1:1,000), anti-p-Src (Cell Signaling, Cat. no. 2101; 1:1,000), anti-Src (Cell Signaling, Cat. no. 2109; 1:1,000), anti-p-FAK (Cell Signaling, Cat. no. 8556; 1:1,000), anti-p-FAK (Cell Signaling, Cat. no. 3283; 1:1,000), anti-FAK (Cell Signaling, Cat. no. 71433; 1:1,000), or anti-MAFG (GeneTex, Cat. no. GTX114541; 1:1,000). Horseradish peroxidase-conjugated secondary antibodies were followed by enhanced chemiluminescence (Thermo Fisher, MA).

### ChIP-qPCR

ChIP was carried out using a Diagenode (Belgium) iDeal ChIP-seq kit for transcription factor in accordance with the manufacturer’s instructions. In brief, 1 × 10^7^ cells were cross-lined with 1% formaldehyde for 8 min at room temperature. Cells were washed, lysed, and sheared by sonication after terminating the fixation with 0.125 M glycine. The sheared chromatin fragments were immunoprecipitated with protein-A-coated magnetic beads and anti-YAP (Cell Signaling, Cat. no. 14074; 1:50), anti-MAFG (GeneTex, Cat. no. GTX114541; 1:50), or anti-IgG (Cell Signaling, Cat. no. 3900; 1:50) antibody. After elution and decross-linking, the enriched DNA fragments were purified by IPure beads v2. The primer sequences used for ChIP-qPCR are described in Table S4.

### Stably overexpressing ITGB8, MAFG, and RhoGDI

ITGB8, MAFG, and RhoGDI WT or mutant cDNAs were inserted into a lentiviral vector plasmid pLV-EF1α-IRES-Puro (Addgene, #85132) with a C-terminal 3×Flag tag for transient expression in 293T cells to obtain the lentivirus containing the target gene. Then, these infected cells were cultured with 1.5 μg/ml puromycin to select cell clones for 4 days. Western blot results verified that target gene overexpression was effective.

### Animal experiments and treatment protocol

MCF-7 or 4T1 cells were injected into the mammary fat pads of BALB/c or NSG mice for tumor growth, while B16 or MP-1 cells were injected subcutaneously into C57BL/6 or NSG mice. Every day, tumors in mice were checked using palpation and observation. The proportion of tumor development was estimated 8 to 12 weeks later. In several studies, the 90-Pa soft 3D fibrin gel with 10 B16 or MCF-7 cells implanted in it was subsequently injected under the skin of NSG mice.

### Bioinformatics analysis

The R program was used to do all analyses, and it was updated from version 3.6.2. For the purposes of statistical analysis and graphical work, R language was employed. According to the R package survival, Kaplan–Meier survival curves and the log-rank test were used to assess the outcomes of patients in the TCGA cohort with MAFG expression. We calculated stemness single-sample Gene Set Enrichment Analysis (ssGSEA) signatures using the Gene Set Variation Analysis (GSVA) package in R according to previous reports [61]. To analyze the correlation between *MAFG* and stemness genes, the R package ggplot2 was used to analyze the TCGA cohort.

### Statistics and reproducibility

We performed at least 3 biological repeats for all experiments except as indicated in the figure legends, and no statistical method was used to predetermine sample size. No data were excluded from analyses. The mice were randomly assigned to different groups. The analysis was conducted using the GraphPad Prism 8.0 software. Results are expressed as mean ± SD as indicated, and analyzed by Student’s *t* test followed by 2-tailed paired *t* test or one-way ANOVA followed by Bonferroni as indicated. *P* value < 0.05 was considered statistically significant. The Spearman’s correlation test was used to examine the relationship between the amount of MAFG, fibrinogen, and cancer grade.

## Data Availability

The article or the Supplementary Materials include all the information required to assess the findings. The source data for this paper are supplied. The RNA-Seq data produced in this study have been deposited in the National Genomics Data Center (NGDC) under accession code HRA003140. Previously published sequencing data that were reanalyzed here are available under accession codes GSE98394 and GSE42568. All other data supporting the findings of this study are available from the corresponding author on reasonable request.
