# Supplementary material for: YAP Inactivation by Soft Mechanotransduction Relieves MAFG for Tumor Cell Dedifferentiation
Source: Research (Wash D C). 2023 Aug 22;6:0215. doi: 10.34133/research.0215 (PMC10443527; doi:10.34133/research.0215)
Supplement: Supplementary 1 — Supplementary Materials and Methods Figs. S1 to S8 Tables S1 to S7 [file research.0215.f1.zip › Supplementary Information-Res-v4.docx]

**Supplementary Materials for**

**YAP inactivation by soft mechanotransduction relieves MafG for tumor cell dedifferentiation**

Jiadi Lv^1,5^, Xiaohan Liu^2,5^, Yabo Zhou^1^, Feiran Cheng^1^, Haoran Chen^1^, Shunshun Li^3^ Dianheng Wang^1^, Li Zhou^1^, Zhenfeng Wang^1^, Nannan Zhou^1^, Jie Chen^1^, Bo Huang^1,4*^

^1^Department of Immunology & State Key Laboratory of Common Mechanism Research for Major Diseases, Institute of Basic Medical Sciences, Chinese Academy of Medical Sciences & Peking Union Medical College (PUMC), Beijing 100005, China

^2^Department of Histology and Embryology, Basic Medical College, China Medical University, Shenyang, Liaoning, 110122, China

^3^Department of Immunology, Basic Medical College, China Medical University, Shenyang, Liaoning, 110122, China

^4^Department of Biochemistry & Molecular Biology, Tongji Medical College, Huazhong University of Science & Technology, Wuhan 430030, China

^5^These authors contributed equally

Running title: Integrin β8 regulates tumor cell dedifferentiation

*Corresponding author: [tjhuangbo@hotmail.com](mailto:tjhuangbo@hotmail.com)

**This file includes:**

Supplementary Materials and Methods

Figs. S1-8

Tables S2-7

**Supplementary Materials and Methods**

**Co-immunoprecipitation (IP) assay**

Cells were lysed in IP buffer (Beyotime, China) and then centrifuged at 20,000 g for 10min. Sample proteins were incubated with anti-ITGB8 antibody (Sigma-Aldrich, Cat.: WH0003696M1) or anti-YAP antibody (Cell Signaling, Cat.: 14074) at 4 °C for 8 hours. Samples were washed with IP buffer 3 times and were incubated with in Protein-A Agarose for 4 hours. Immunoprecipitants were washed with IP buffer 3 times and boiled in SDS sample buffer for 8 min. Then, the Immunoprecipitants were run on an SDS-PAGE gel.

**Real-Time PCR**

Total RNA was extracted from cells using Trizol (Invitrogen) and was transcribed to cDNA by using a High-Capacity cDNA Reverse Transcription Kit (Applied Biosystems, CA). The primer sequences were described in Table S5. Real-time PCR was performed using ABI QuantStudio (Applied Biosystems, CA, USA). Values are means ± SD from three independent experiments which were performed in duplicate.

**Immunofluorescence**

Cells were fixed in 4 % paraformaldehyde and permeabilized with 0.2 % Triton X-100. Fixed cells were blocked in 5 % BSA and incubated with anti-YAP (Cell Signaling, Cat.: 14074; 1:200) or anti-MAFG (GeneTex, Cat.: GTX114541; 1:200) at 4℃ overnight. Then, cells were washed and incubated with secondary antibodies for 2 hours at room temperature. Finally, the slides were counterstained with DAPI and mounted for confocal analysis. The intensity of immunofluorescence was analyzed by Image J software.

**Histological and immunohistochemical staining**

The tumor tissues from mice or melanoma or breast cancer patients were fixed in 10% formalin, embedded in paraffin and sectioned for H&E staining. Immunohistochemical staining was performed using the DAB Horseradish Peroxidase Color Development Kit (ZSGB-BIO, China) according to the manufacturer’s instructions. Briefly, the sections of paraffin embedded tissues were incubated with anti-MAFG (GeneTex, Cat.: GTX114541; 1:200) or anti-Fibrinogen (Abcam, Cat.: ab34269; 1:200) at 4̊C overnight. Afterwards, slides were sequentially incubated with HRP-conjugated secondary antibodies for one hour at room temprature, followed by developing with DAB substrate and counterstaining with hematoxilin. The stained sections were scanned and digitalized utilizing a Pannoramic Midi II (3D Histech, Hungary). The intensity of positive staining was analyzed by Image J 9.0 software.

**Construction of the SOX2- and BCL9L-GFP reporter expression plasmid**

A genomic fragment derived from the promoter (−2000 ∼ +1) of the human SOX2, human BCL9L, mouse Sox2 or mouse Bcl9l gene was PCR amplified and subsequently cloned into pLVX-DD-ZsGreen1 Reporter vector (Clontech, USA). The DNA sequences of the genomic fragment obtained by PCR were verified using automated DNA sequencing.

**Generation of CRISPR-Cas9 knockout cell lines.**

For construction of the stable knockout of *ITGB8*, *RhoGDI, YAP, LATS1, LATS2* or *MAFG*-B16 or MCF-7 cells, the following SGRNAs targeting *ITGAV, ITGB8*, *RhoGDI, YAP* or *MAFG* were were described in Table S6. These SGRNAs were cloned into the px459 vector plasmid (Addgene, #48139) and transfected B16 or 4T1 cells. 48 hr later, B16 or 4T1 cells were treated with puromycin at a final concentration of 2 μg/ml. After 48 hr, puromycin-resistant cells were seeded in 96-well cell plates. The candidate knockout cells were verified by western blot.

**siRNA-mediated gene silencing**

B16 or MCF-7 cells were plated in 6-well plates (at a density of 2× 10^5^ cells per well) and then were transfected with 20 μM siRNA according to the manufacturer’s guidelines. GenePharma chemically synthesized siRNA sequences, and the negative control siRNA was also from GenePharma. The siRNA sequences were described in Table S7.

**Flow cytometry**

The tumor tissues were digested collagenase type Ⅳ (Sigma, Cat.:C5138; 1 mg/ml) and DNase Ⅰ (Sigma, Cat.:11284932001; 5 μg/ml). After the digestion, single-cell suspensions were obtained by filtering through a 70 µm cell strainer before being pelleted by centrifugation and then erythrocytes (RBC) were removed by exposed to RBC lysis buffer. All samples were stained with Live/Dead dye (Biolegend, Zombie Aqua Fixable Viability Kit, 100×) for 15min at room temperature before they were stained with PE anti-mouse CD45 antibody (Biolegend, Cat.:147712; 1:100). Invitrogen Attune NxT software was used for data collection. Data were analyzed with FlowJo software.

**Supplementary Figures**


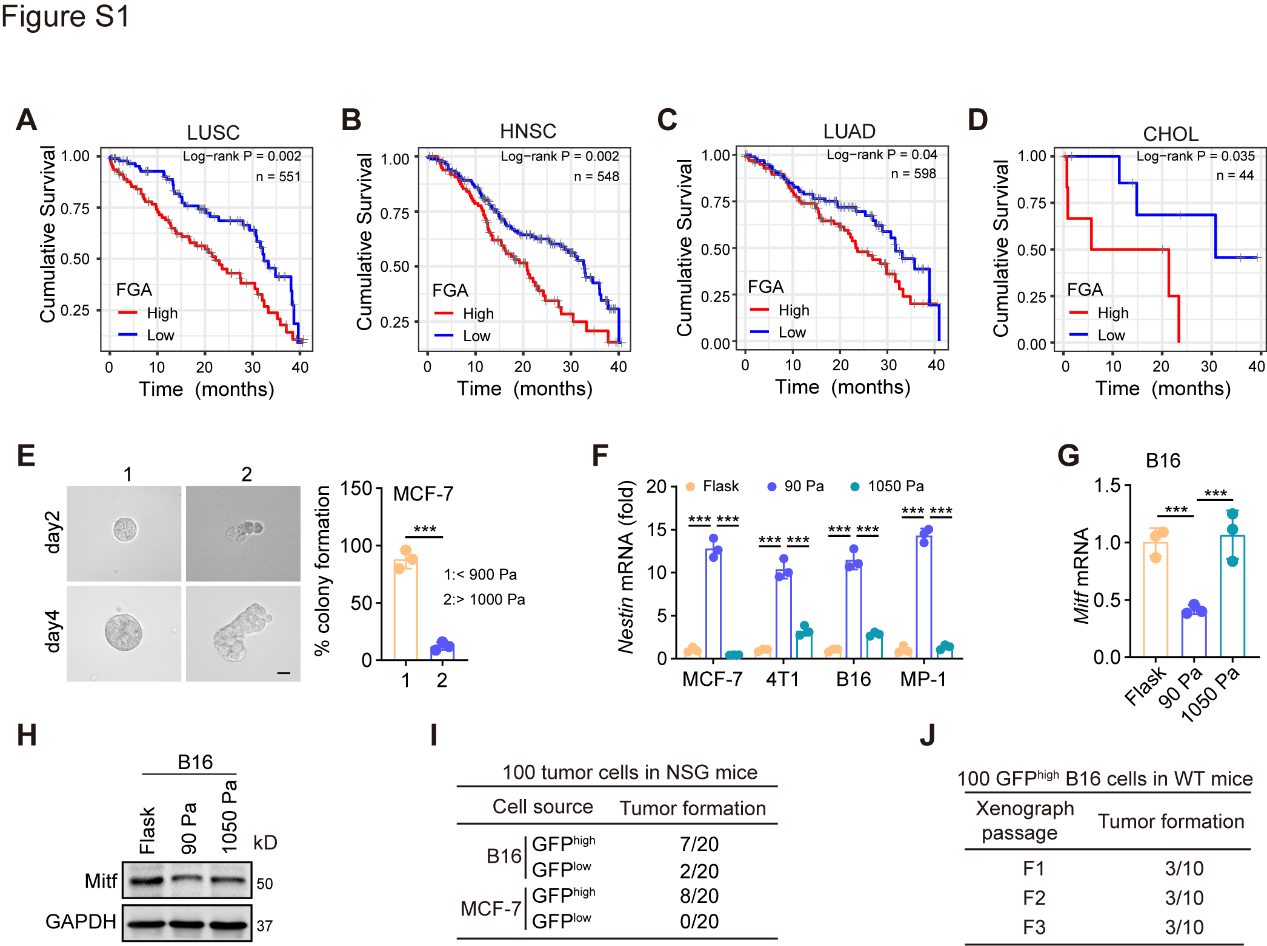


**Fig. S1 The moderately stiff tumor cells can undergo dedifferentiation in a soft mechanical microenvironment.** (**A-D**) Kaplan–Meier analysis of fibrinogen alpha chain (*FGA*) expression based on overall survival (OS) in Lung squamous cell carcinoma (LUSC, **A**), Head and Neck squamous cell carcinoma (HNSC, **B**), Lung adenocarcinoma (LUAD, **C**) and Cholangio carcinoma (CHOL, **D**) cohorts in TCGA database. (**E**) The different stiffness of MCF-7 cells (100 cells per group) were seeded in 90 Pa soft 3D fibrin gel. The percentage of colony formation was calculated. Scale bar, 100 μm. (**F**) The mRNA expression of *Nestin* in B16,4T1, MCF-7 and MP-1 tumor cells cultured in 90 Pa 3D fibrin gel or flask for 48 hr was analyzed by qPCR. (**G** and **H**) The levels of Mitf protein in B16 tumor cells cultured in 90 Pa, 1050 Pa 3D fibrin gel or on flask for 48 hr were analyzed by qPCR (**G**) or western blot (**H**). (**I**) 100 GFP^low^ or GFP^high^ tumor cells from *BCL9L* promotor-GFP expressing B16 or MCF-7 cells were inserted into the subcutaneous site of NSG. Eight weeks later, the tumor formation was recorded. (**J**) The tumor‐forming capacity from primary xenografts (F1) and tumors passaged into secondary (F2) and tertiary (F3) recipients induced by injecting 100 GFP^high^ B16 cells into WT mice. n = 10. In **E**, **F** and **G**, n = 3 biological independent experiments. ****P*<0.001, by two-tailed Student’s t-test (**E**) or one-way ANOVA Bonferroni's test (**F** and **G**). The data represent mean ± SD.


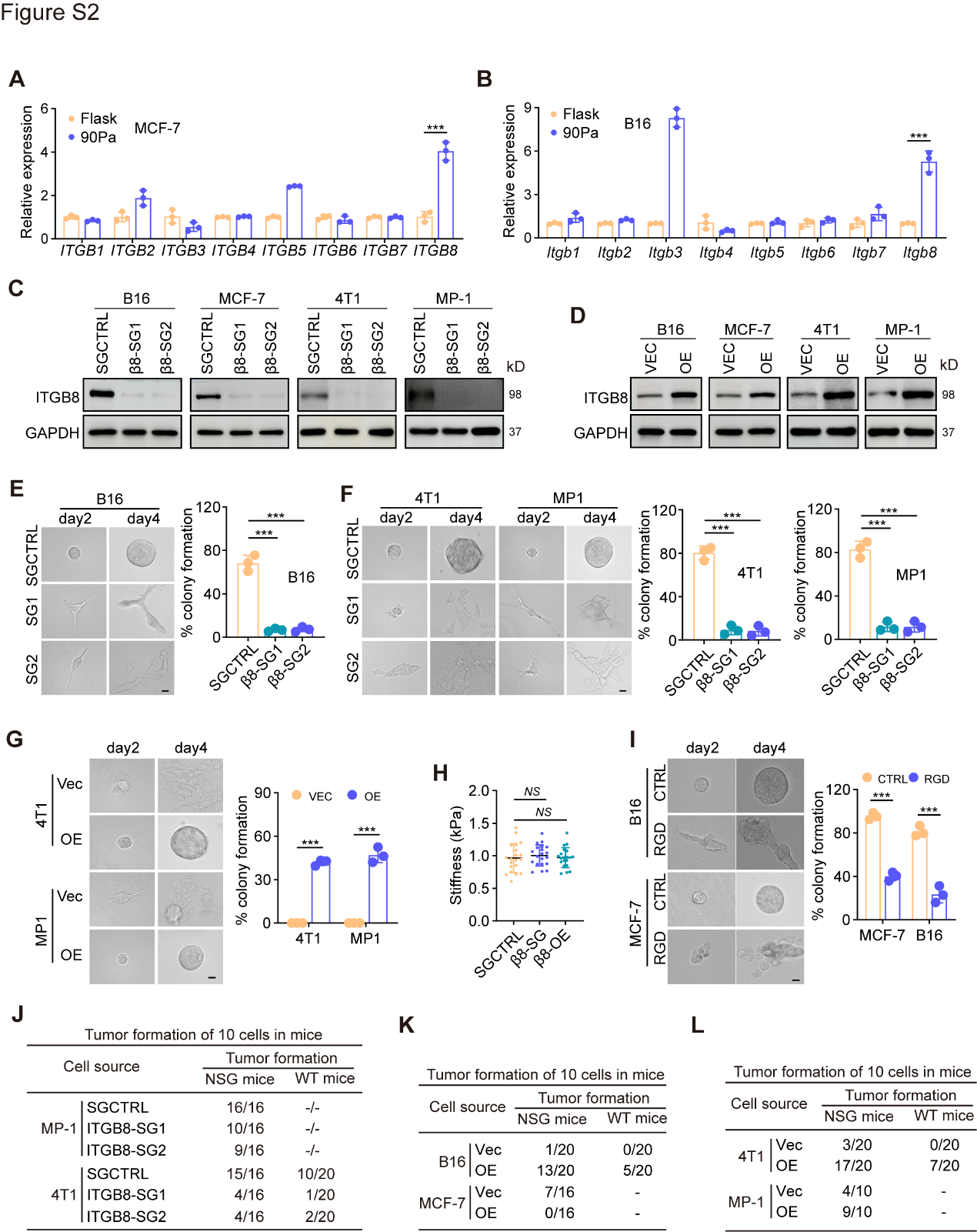
**Fig. S2 Integrin β8 mediates the tumor cell dedifferentiation.** (**A** and **B**) The mRNA expression of *ITGB1-ITGB8* in MCF-7 (**A**) or B16 (**B**) tumor cells cultured in 90 Pa soft 3D fibrin gel or flask was analyzed by qPCR. (**C** and **D**) The knockout (**C**) or overexpression (**D**) efficiency of ITGB8 in B16, MCF-7, 4T1 or MP-1 cells was determined by western blot. (**E**) SGCTRL or *ITGB8*-SGs B16 cells were seeded in 90Pa soft 3D fibrin gel for 4 days. The colonies were presented photographically (left) and the percentage of colony formation was calculated (right). Scale bar, 20 μm. (**F**) The same as (**E**), except that 4T1 or MP-1 cells. (**G**) The same as (**E**), except that H-stiff vector or *ITGB8*-OE-4T1 or MP-1 cells. Scale bar, 20 μm. (**H**) The stiffness of SGCTRL, *ITGB8*-SG or *ITGB8*-OE-B16 tumor cells cultured on rigid plate (Flask) for 48 h was measured by AFM. n = 20. (**I**) B16 or MCF-7 cells were seeded in 90Pa soft 3D fibrin gel treated with or without RGD peptide (50μM) for 4 days. The colonies were presented photographically (left) and the percentage of colony formation was calculated (right). Scale bar, 20 μm. (**J**) 10 M-stiff SGCTRL or *ITGB8*-SGs-4T1 or MP-1 tumor cells embedded in 50 μl fibrin gel (90 Pa) were inserted into the subcutaneous site of WT or NSG mice (n=20 or n=16). The tumor formation was recorded. (**K** and **L**) The same as (**J**), except that H-stiff vector or *ITGB8*-OE-B16, MCF-7, 4T1 or MP-1 cells. In **A, B, E-G** and **I**, n = 3 biological independent experiments. ****P*<0.001, by two-tailed Student’s t-test (**A, B, G** and **I**) or one-way ANOVA Bonferroni's test (**E, F** and **H**). The data represent mean ± SD.


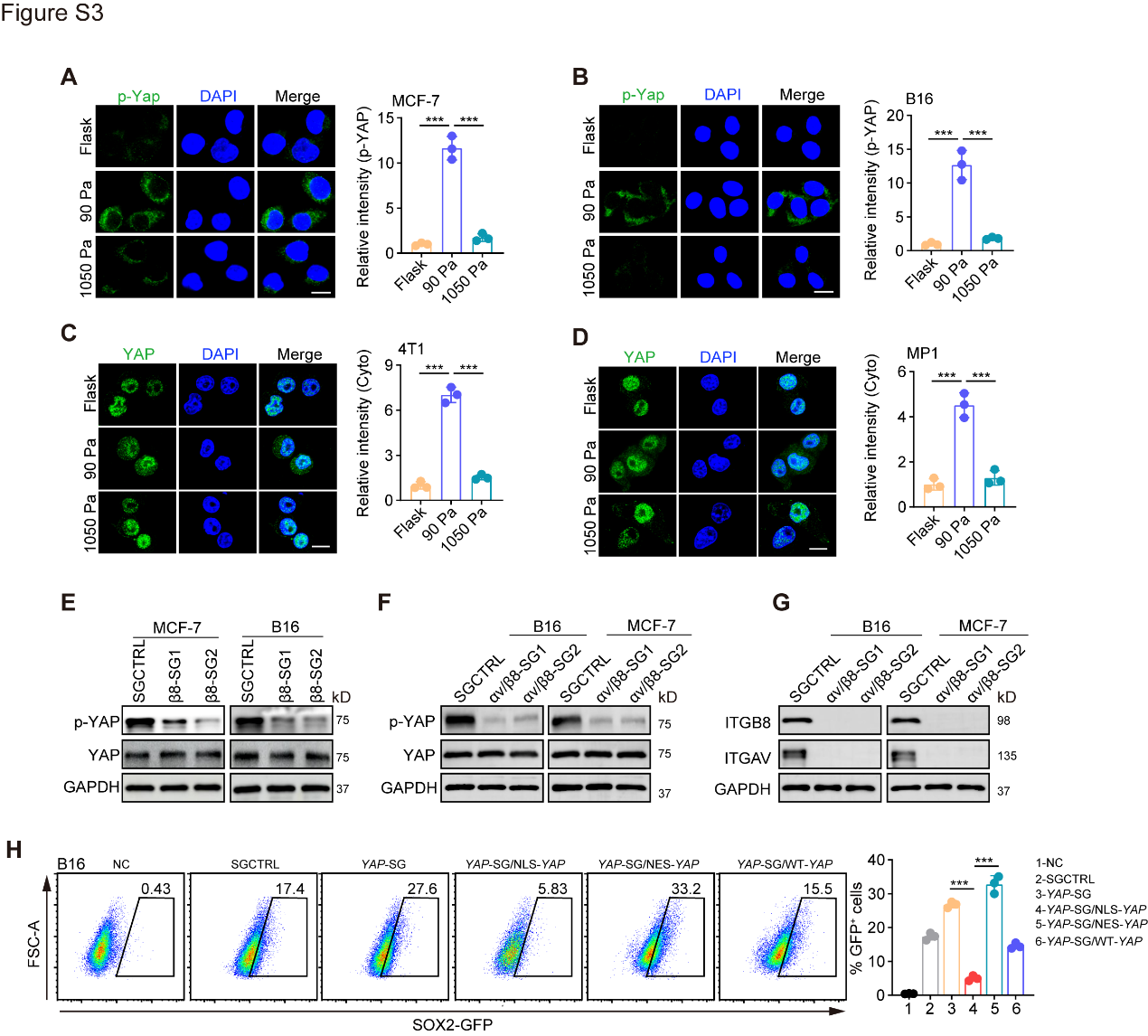


**Fig. S3 Integrin β8 mediates YAP inactivation in a soft mechanical microenvironment.** (**A** and **B**) Immunostaining of P-YAP in MCF-7 (**A**) or B16 (**B**) cells cultured in flask, 90 Pa or 1050 Pa 3D fibrin gel for 48 hr. Scale bars, 10 μm. (**C** and **D**) Immunostaining of YAP in 4T1 (**C**) or MP-1 (**D**) cells cultured in flask, 90 Pa or 1050 Pa 3D fibrin gel for 48 hr. Scale bars, 10 μm. (**E**) The levels of p-YAP and total YAP protein in SGCTRL or *ITGB8*-SGs-B16 or MCF-7 cells were seeded in 90 Pa soft 3D fibrin gel for 48 hr were analyzed by western blot. (**F**) The levels of p-YAP and total YAP protein in SGCTRL or αv/β8-SGs-B16 or MCF-7 cells were seeded in 90 Pa soft 3D fibrin gel for 48 hr were analyzed by western blot. (**G**) The knockout efficiency of αv/β8 in B16 or MCF-7 cells was determined by western blot. (**H**) SGCTRL, *YAP*-SG, *YAP*-SG/NLS-*YAP*, *YAP*-SG/NES-*YAP* or *YAP*-SG/WT-*YAP*-B16 cells which were Sox2 promotor-GFP expressing were seeded in 90Pa soft 3D fibrin gel for 48 hr. The expression of GFP in single B16 cell was analyzed by flow cytometry. n = 3. In **A**-**D**, n = 3 biological independent experiments. ****P*<0.001, by one-way ANOVA Bonferroni's test (**A**-**D** and **H**). The data represent mean ± SD.


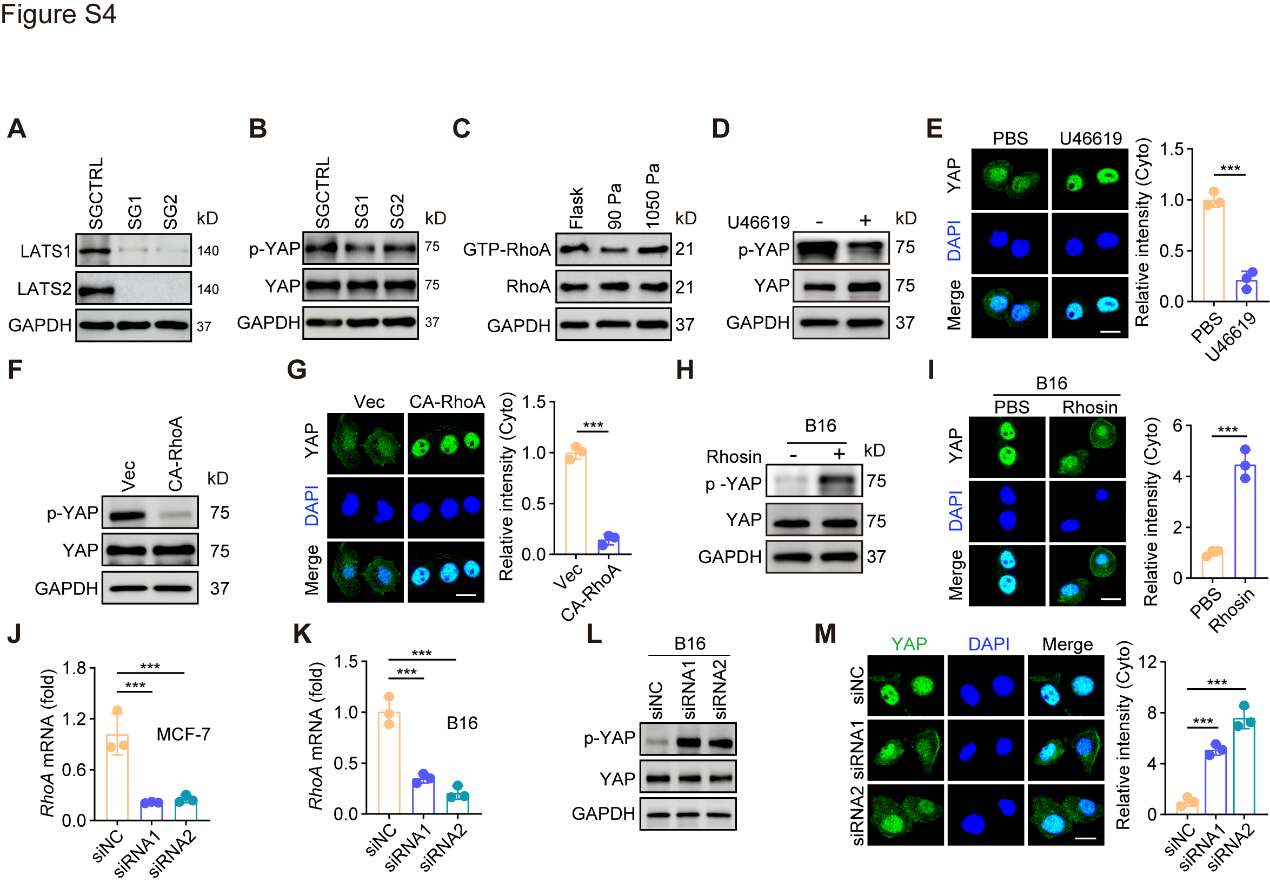


**Fig. S4 Integrin β8-imediated YAP inactivation relies on RhoA inactivation.** (**A**) The knockout efficiency of LATS1 and LATS2 in MCF-7 cells was determined by western blot. (**B**) The levels of p-YAP1 or total YAP in SGCTR or LATS1/2-SGs-MCF-7 cells were analyzed by western blot. (**C**) The levels of GTP-RhoA or total RhoA protein in B16 cells cultured on rigid plate, in 90 Pa or 1050 Pa 3D fibrin gel for 48 hr were analyzed by western blot. (**D**) The levels of p-YAP or total YAP protein in B16 cells treated with U46619 (1 μM) when cultured in 90 Pa soft 3D fibrin gel for 48hr were analyzed by western blot. (**E**) Immunostaining of YAP in B16 cells treated with U46619 (1μM) when cultured in 90 Pa soft 3D fibrin gel for 48hr. Scale bar, 10 μm. (**F**) The levels of p-YAP or total YAP protein in B16 cells which were transfected with constitutively active RhoA (Q63L) (CA-RhoA) or vector cultured in the 90 Pa soft fibrin gels for 48 hr were analyzed by western blot. (**G**) Immunostaining of YAP in B16 cells which were transfected with CA-RhoA or vector cultured in 90 Pa soft 3D fibrin gel for 48 hr. Scale bar, 10 μm. (**H**) same as (D), except tumor cells treated with Rhosin (10 μM). (**I**) same as (E), except tumor cells treated with Rhosin (10 μM) and cultured in 1050 Pa soft 3D fibrin gel for 48 hr. Scale bar, 10 μm. (**J** and **K**) The knockdown efficiency of RhoA in MCF-7 (**J**) or B16 (**K**) cells was analyzed by qPCR. (**L**) The levels of total YAP1 or p-YAP in B16 cells which were transfected with RhoA siRNA or siNC cultured in 1050 Pa 3D fibrin gel for 48 hr was analyzed by western blot. (**M**) Immunostaining of YAP in B16 cells which were transfected with RhoA siRNAs or siNC cultured in 90 Pa soft 3D fibrin gel. Scale bar, 10 μm. In **E, G, I, J, K** and **M**, n = 3 biological independent experiments. ****P*<0.001, by two-tailed Student’s t-test (**E, G** and **I**) or one-way ANOVA Bonferroni's test (**J, K** and **M**). The data represent mean ± SD.


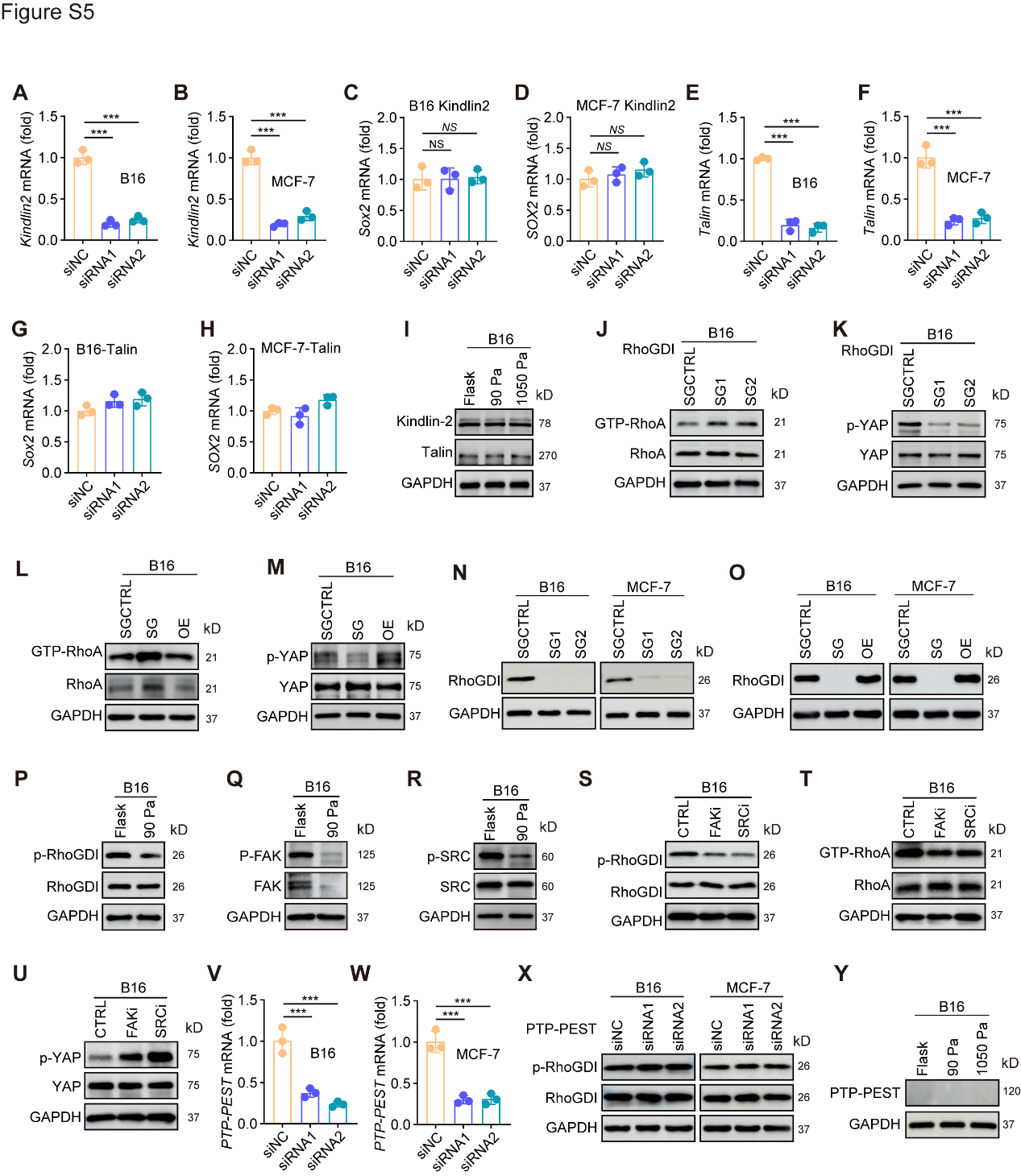


**Fig. S5 RhoGDI recruited by Integrin β8 suppresses the activity of RhoA.** (**A** and **B**) The knockdown efficiency of *Kindlin2* in B16 (**A**) or MCF-7 (**B**) cells was determined by qPCR. (**C** and **D**) The expression of *Sox2* in B16 (**C**) or MCF-7 (**D**) cell transfected with *Kindlin2* siRNAs was analyzed by qPCR. (**E** and **F**) The knockdown efficiency of *Talin* in B16 (**E**) or MCF-7 (**F**) cells was determined by qPCR. (**G** and **H**) The same as (**E** and **F**), except B16 (**G**) or MCF-7 (**H**) cells were transfected with *Kindlin2* siRNAs. (**I**) The levels of Kindlin2 and Talin in B16 cells cultured in Flask, 90 Pa or 1050 Pa 3D fibrin gel for 48 hr were analyzed by western blot. (**J** and **K**) The levels of GTP-RhoA, total RhoA (J), p-YAP or total YAP (K) in SGCTRL or *RhoGDI*-SGs-B16 tumor cells were analyzed by western blot. (**L** and **M**) same as (J, K), except the SGCTRL, *RhoGDI*-SG or *RhoGDI*-SG*/RhoGDI*-OE B16 tumor cells. (**N** and **O**) The knockout (N) or overexpression (O) efficiency of RhoGDI in B16 or MCF-7 cells was determined by western blot. (**P**) The levels of p-RhoGDI or total RhoGDI in B16 cells cultured in Flask or 90 Pa 3D fibrin gel for 48 hr were analyzed by western blot. (**Q** and **R**) The levels of p-FAK, FAK (Q), p-SRC or SRC (R) in B16 cells cultured in Flask or 90 Pa 3D fibrin gel for 48 hr were analyzed by western blot. (**S-U**) The levels of p-RhoGDI (S), RhoGDI (S), GTP-RhoA (T), RhoA (T), p-YAP (U) or YAP (U) in B16 cells treated with PBS, SRC inhibitor (Dasatinib, 5 μM) or FAK inhibitor (GSK2256098, 5 μM) for 48 hr were analyzed by western blot. (**V** and **W**) The knockdown efficiency of *PTP-PEST* in B16 (**V**) or MCF-7 (**W**) cells was determined by qPCR. (**X**) The levels of RhoGDI or p-RhoGDI in MCF7 or B16 tumor cells transfected with PTP-PEST siRNAs were analyzed by western blot. (**Y**) The level of PTP-PEST in B16 cells cultured in Flask, 90 Pa or 1050 Pa 3D fibrin gel was analyzed by western blot. In **A**-**H**, **V** and **W**, n = 3 biological independent experiments. ****P*<0.001, by one-way ANOVA Bonferroni's test (**A**-**H**, **V** and **W**). *NS*, no significant difference. The data represent mean ± SD.


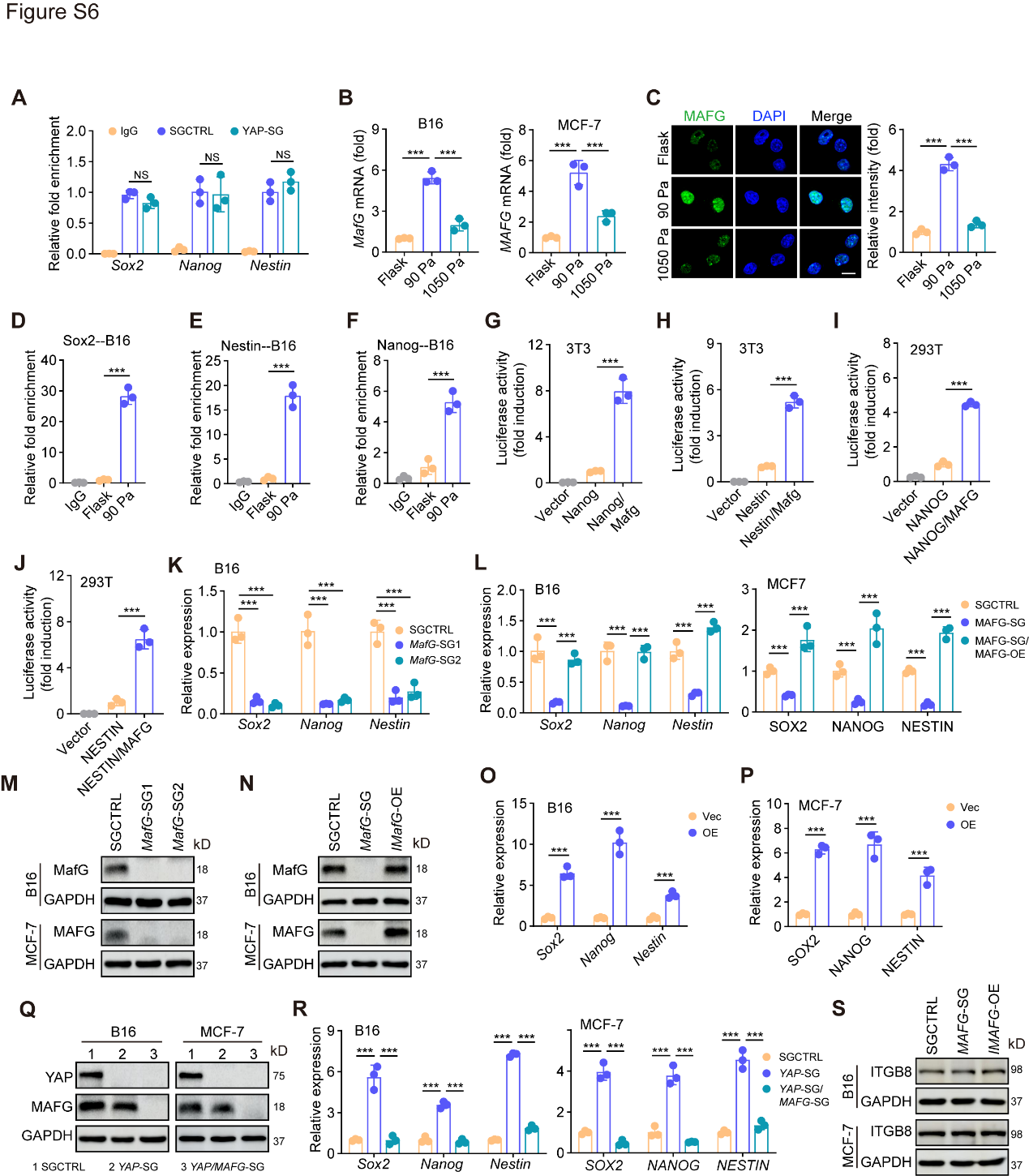


**Fig. S6 Inactivated YAP promotes the transcription of MAFG.** (**A**) ChIP-qPCR analysis was performed with an antibody to YAP1 and *Sox2*, *Nestin* or *Nanog* promotor-specific primers in SGCTRL or *YAP1*-SG-B16 cells cultured in 90 Pa 3D fibrin gel. (**B**) The levels of MafG protein in B16 or MCF-7 cells cultured in 90 Pa, 1050 Pa 3D fibrin gel or on flask for 48 hr were analyzed by qPCR. (**C**) Immunostaining of of MafG in B16 tumor cells cultured in 90 Pa, 1050 Pa 3D fibrin gel or on flask for 48 hr. (**D-F**) ChIP-qPCR analysis was performed with an antibody to MafG and *Sox2* (**D**), *Nestin* (**E**) or Nanog (**F**) promotor-specific primers in B16 cells cultured on rigid plate or in 90 Pa 3D fibrin gel. (**G** and **H**) The NIH3T3 cells were co-transfected with *Nanog* (**G**) or *Nestin* (**H**) promoter-luciferase reporter PGL3 and pCMV-MAFG plasmid for 24hr followed by analysis of luciferase activity. (**I** and **J**) The same as (**G** and **H**), except the cells were HEK293T. (**K**) The expression of *Nestin*, *Nanog* and *Sox2* in SGCTRL or *MafG*-SGs-B16 cultured in 90 Pa 3D fibrin gel was analyzed by qPCR. (**L**) The expression of *NESTIN*, *NANOG* and *SOX2* in SGCTRL or *MAFG*-SG-B16 or MCF-7 cells with or without MAFG overexpression was detected by real-time PCR. (**M** and **N**) The knockout (**M**) or overexpression (**N**) efficiency of MAFG in B16 or MCF-7 cells was determined by western blot. (**O** and **P**) The expression of stemness genes in vector or MafG-overexpressed B16 (**O**) or MCF-7 (**P**) cells cultured on rigid plate. (**Q**) The knockout efficiency of YAP or MAFG in B16 or MCF-7 cells was determined by western blot. (**R**) The expression of SOX2 in SGCTRL, *YAP*-SG or *MAFG/YAP*-SG-B16 (left) or MCF-7 (right) cells cultured in 90 Pa 3D fibrin gel was analyzed by qPCR. (**s**) The level of ITGB8 in SGCTRL, *MAFG*-SG or *MAFG*-SG*/ MAFG*-OE B16 (up) or MCF-7 (down) cells cultured in 90 Pa 3D fibrin gel was analyzed by western blot. In **A**-**L**, **O**, **P** and **R**, n = 3 biological independent experiments. ****P*<0.001, by one-way ANOVA Bonferroni's test (**A**-**L** and **R**) and Student’s T test (**O** and **P**). The data represent mean ± SD.


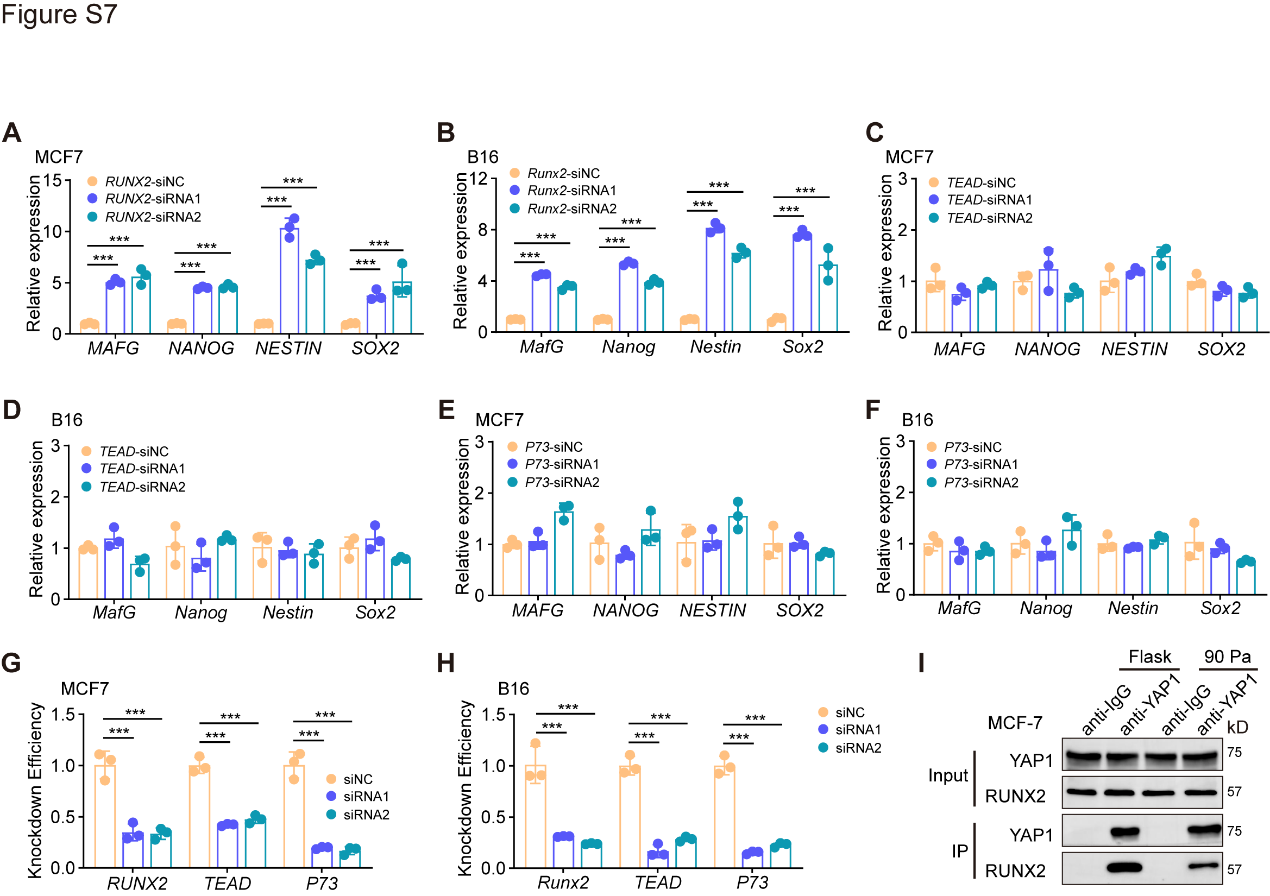


**Fig. S7 YAP was binding with Runx2 to repress the transcription of MafG.** (**A** and **B**) The expression of *MAFG, NANOG, NESTIN* and *SOX2* in MCF-7 (**A**) or B16 (**B**) cell transfected with *Runx2* siRNAs cultured on flask for 48 hr was analyzed by qPCR. (**C** and **D**) The same as (**A** and **B**), except cells were transfected with *TEAD* siRNAs. (**E** and **F**) The same as (A and B), except cells were transfected with *P73* siRNAs. (**G** and **H**) The knockdown efficiency of *RUNX2, TEAD OR P73* in MCF-7 (**G**) or B16 (**H**) cells was determined by qPCR. (**I**) MCF-7 cells were cultured on Flask or into 90 Pa 3D fibrin gel for 48 hr. Cell lysates were collected for immunoprecipitation with anti-YAP or anti-IgG antibody. The expression of RUNX2 was analyzed by western blot. In **A**-**H**, n = 3 biological independent experiments. ****P*<0.001, by one-way ANOVA Bonferroni's test (**A**-**H**). The data represent mean ± SD.


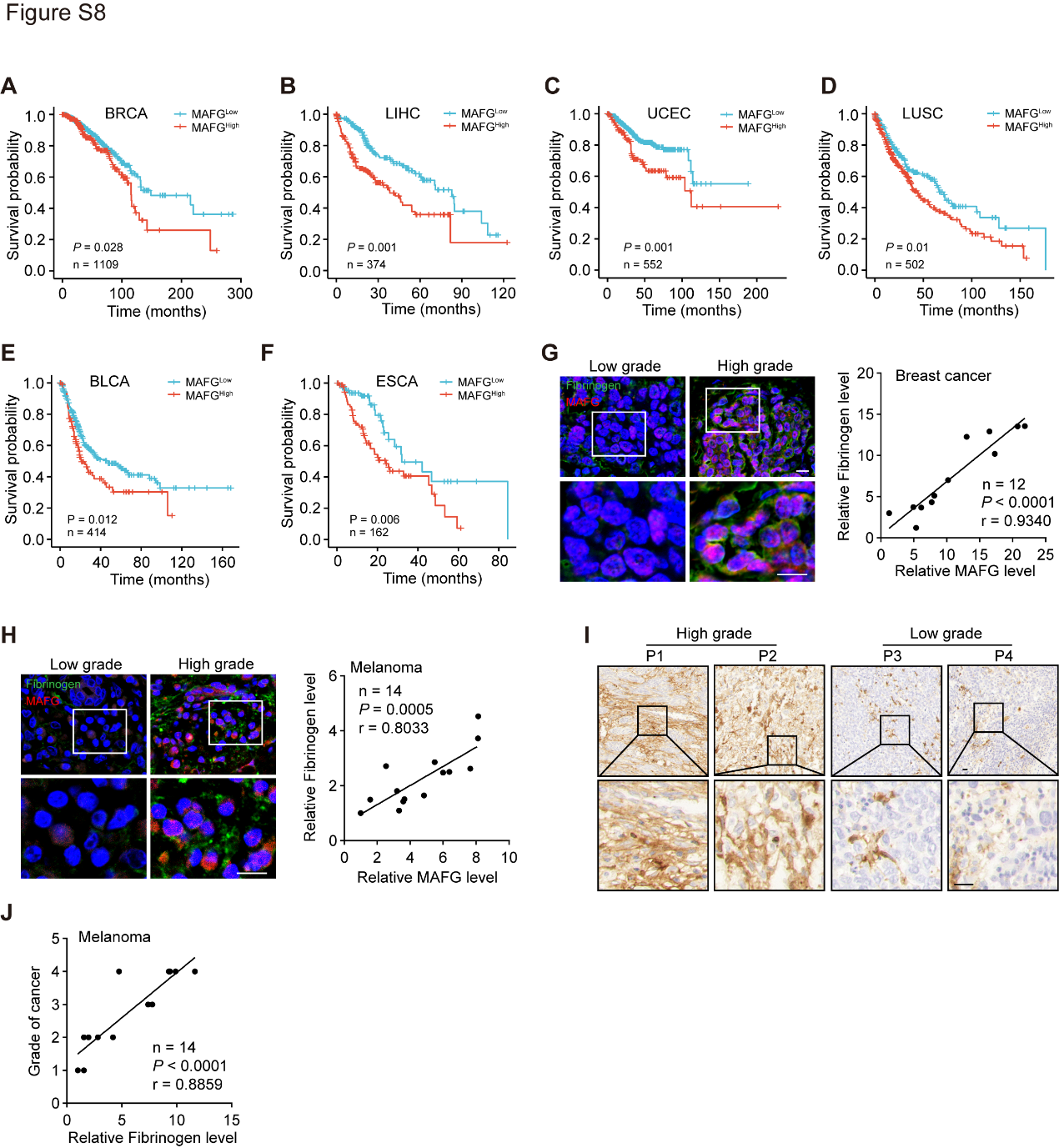
**Fig. S8 MafG signature is correlated with higher tumor pathological grade and worse prognosis.** (**A-F**) Overall survival compared with the MAFG level in patients with breast cancer (BRCA, **A**), liver hepatocellular carcinoma (LIHC, **B**), uterine corpus endometrial carcinoma (UCEC, **C**), lung squamous cell carcinoma (LUSC, **D**) bladder urothelial carcinoma (BLCA, **E**) or esophageal carcinoma (ESCA, **F**). (**G**) Immunostaining of MAFG and Fibrinogen in the tumor tissue sections from patients with low or high grade of BRCA (left). The correlation between the expression of MAFG and Fibrinogen in tumor tissues from 12 patients with breast cancer was showed (right). Scale bars, 10 μm.

(**H**) Immunostaining of MAFG and Fibrinogen in the tumor tissue sections from patients with low or high grade of melanoma (left). The correlation between the expression of MAFG and Fibrinogen in tumor tissues from 14 patients with melanoma was showed (right). Scale bars, 10 μm. (**I** and **J**) The tissue sections from 2 melanoma patients with high grade of malignancy (I, left) and 2 melanoma patients with low grade of malignancy (I, right) were immunohistochemical staining with anti-Fibrinogen antibody. The correlation between the expression of Fibrinogen from 14 patients with melanomar and tumor pathological grade was showed (J). Scale bars, 50 μm. Results are analyzed by by Log-rank survival analysis (**A**-**F**) and Spearman’s correlation test (**G, H** and **J**).

**Table S2 Clinical information of melanoma patients**

| **Patients NO.** | **Gender** | **Age** | **Status** | **Sample** |
| --- | --- | --- | --- | --- |
| 1 | Female | 55 | Newly diagnosis | Tumor tissue |
| 2 | Male | 67 | Newly diagnosis | Tumor tissue |
| 3 | Female | 50 | Newly diagnosis | Tumor tissue |
| 4 | Male | 48 | Newly diagnosis | Tumor tissue |
| 5 | Female | 64 | Newly diagnosis | Tumor tissue |
| 6 | Female | 56 | Newly diagnosis | Tumor tissue |
| 7 | Female | 62 | Newly diagnosis | Tumor tissue |
| 8 | Male | 60 | Newly diagnosis | Tumor tissue |
| 9 | Male | 69 | Newly diagnosis | Tumor tissue |
| 10 | Male | 61 | Newly diagnosis | Tumor tissue |
| 11 | Male | 63 | Newly diagnosis | Tumor tissue |
| 12 | Female | 65 | Newly diagnosis | Tumor tissue |
| 13 | Male | 43 | Newly diagnosis | Tumor tissue |
| 14 | Male | 69 | Newly diagnosis | Tumor tissue |

**Table S3 Clinical information of breast cancer patients**

| **Patients NO.** | **Gender** | **Age** | **Status** | **Sample** |
| --- | --- | --- | --- | --- |
| 1 | Female | 63 | Newly diagnosis | Tumor tissue |
| 2 | Female | 70 | Newly diagnosis | Tumor tissue |
| 3 | Female | 80 | Newly diagnosis | Tumor tissue |
| 4 | Female | 42 | Newly diagnosis | Tumor tissue |
| 5 | Female | 50 | Newly diagnosis | Tumor tissue |
| 6 | Female | 60 | Newly diagnosis | Tumor tissue |
| 7 | Female | 37 | Newly diagnosis | Tumor tissue |
| 8 | Female | 69 | Newly diagnosis | Tumor tissue |
| 9 | Female | 72 | Newly diagnosis | Tumor tissue |
| 10 | Female | 59 | Newly diagnosis | Tumor tissue |
| 11 | Female | 62 | Newly diagnosis | Tumor tissue |
| 12 | Female | 63 | Newly diagnosis | Tumor tissue |

**Table S4 The information of primer sequences used for ChIP-qPCR**

| ChIP-qPCR Primers | |
| --- | --- |
| m*Itgb8* | 5’-GCGGCAAGAGGATGAAATGT-3’ (sense) |
|  | 5’-GTAGTGGCGCAACAGGAATC-3’ (antisense) |
| m*Mafg* | 5’- AACAGCAAATAGGTTCCCGC -3’ (sense) |
|  | 5’- TCTCCACACCAACTCAGACC-3’ (antisense) |
| m*Sox2* | 5’- AGGAGAGAAGTTTGGAGCCC-3’ (sense) |
|  | 5’- TCTGGCGGAGAATAGTTGGG-3’ (antisense) |
| m*Nanog* | 5’-AAACAAACACCACCAACCCC-3’ (sense) |
|  | 5’-ATAAGCAGAGAGGGAAGGGC-3’ (antisense) |
| m*Nestin* | 5’-CAATGTTGCCTCTCTTGGGG-3’ (sense) |
|  | 5’-CAGCCCAGAACACCCTAAGA-3’ (antisense) |
| h*ITGB8* | 5’-ACTGGGAAGGTGACGCTTAA-3’ (sense) |
|  | 5’-GTGTCCCTAGCCTTTGGAGA-3’ (antisense) |
| h*MAFG* | 5’-CAATGCACGACTTCACCCTC-3’ (sense) |
|  | 5’-AGGGGCATGATATGGTCCTG-3’ (antisense) |
| h*SOX2* | 5’-ATCCCCTTTGCTACGGTTGA-3’ (sense) |
|  | 5’-ACCTTCCTTGCTTCCACGTA-3’ (antisense) |
| h*NANOG* | 5’-CACGGCCTCCCAATTTACTG-3’ (sense) |
|  | 5’-GCTGCAGAGTAACCCAGACT-3’ (antisense) |
| h*NESTIN* | 5’-AGGCTGGATAAACTTGGGCT-3’ (sense) |
|  | 5’-CCGTCATGGGCTTAAGAACG-3’ (antisense) |

**Table S5 The information of primer sequences used for Real-Time PCR**

| Real-Time PCRA Primers | |
| --- | --- |
| m*Gapdh* | 5’-AATGGATTTGGACGCATTGGT-3’ (sense) |
|  | 5’-TTTGCACTGGTACGTGTTGAT-3’ (antisense) |
| m*Itgb1* | 5’-TGACCCCAATACCAATCTCCC-3’ (sense) |
|  | 5’- CCTGAAGTGAACTTGTGGCAG-3’ (antisense) |
| m*Itgb2* | 5’-CACTGTCTCAGTTGTGTACCAAG-3’ (sense) |
|  | 5’-GCTCTGGTGTATCACAGCGAA-3’ (antisense) |
| m*Itgb3* | 5’-GAGCCCATTTTCTTCTCCCG-3’ (sense) |
|  | 5’-GCAACACCATGAATCCATCCC -3’ (antisense) |
| m*Itgb4* | 5’-AGAGCTGTACCGAGTGCATC-3’ (sense) |
|  | 5’-TGGTGTCGATCTGGGTGTTCT-3’ (antisense) |
| m*Itgb5* | 5’-GAAGTGCCACCTCGTGTGAA-3’ (sense) |
|  | 5’- GGACCGTGGATTGCCAAAGT-3’ (antisense) |
| m*Itgb6* | 5’-ATGGGGATTGAGCTGGTCTG-3’ (sense) |
|  | 5’-GACAGGTGGGTGAAATTCTCC-3’ (antisense) |
| m*Itgb7* | 5’-ACCTGAGCTACTCAATGAAGGA-3’ (sense) |
|  | 5’-CACCGTTTTGTCCACGAAGG -3’ (antisense) |
| m*Itgb8* | 5’-TGCATGTTGTAACGTCAAGTGA-3’ (sense) |
|  | 5’- GATGCTGACACATCAACCAGATA-3’ (antisense) |
| m*Sox2* | 5’-GCGGAGTGGAAACTTTTGTCC-3’ (sense) |
|  | 5’-GGGAAGCGTGTACTTATCCTTCT-3’ (antisense) |
| m*Nanog* | 5’-CACAGTTTGCCTAGTTCTGAGG-3’ (sense) |
|  | 5’-GCAAGAATAGTTCTCGGGATGAA-3’ (antisense) |
| m*Nestin* | 5’-CCCTGAAGTCGAGGAGCTG-3’ (sense) |
|  | 5’-CTGCTGCACCTCTAAGCGA-3’ (antisense) |
| m*Mafg* | 5’-AGGCGCACACTGAAGAACC-3’ (sense) |
|  | 5’-CCGAGCATCCGTCTTGGAC-3’ (antisense) |
| m*RhoA* | 5’-GAAACTGGTGATTGTTGGTGATG-3’ (sense) |
|  | 5’-ACCGTGGGCACATAGACCT-3’ (antisense) |
| m*Talin* | 5’-TACTACATGCTCCGAAATGGGG-3’ (sense) |
|  | 5’-CACCGTTCCGTCTAACATCCG-3’ (antisense) |
| m*Kindlin2* | 5’-TGGACGGGATAAGGATGCCA-3’ (sense) |
|  | 5’-TGACATCGAGTTTTTCCACCAAC-3’ (antisense) |
| m*Ptp-PEST* | 5’-TCCTGAGGAGGTTCATCCAGA-3’ (sense) |
|  | 5’-CAGTGGCTGTGGGATAAATCTT-3’ (antisense) |
| h*GAPDH* | 5’-TGTGGGCATCAATGGATTTGG-3’ (sense) |
|  | 5’-ACACCATGTATTCCGGGTCAAT-3’ (antisense) |
| h*ITGB1* | 5’-TTGACCCTAATACCAACCTTCCT-3’ (sense) |
|  | 5’-CCCATAGTACAGCCCTTGATGTT-3’ (antisense) |
| h*ITGB2* | 5’-TGCGTCCTCTCTCAGGAGTG-3’ (sense) |
|  | 5’-GGTCCATGATGTCGTCAGCC-3’ (antisense) |
| h*ITGB3* | 5’-GTGACCTGAAGGAGAATCTGC-3’ (sense) |
|  | 5’-CCGGAGTGCAATCCTCTGG-3’ (antisense) |
| h*ITGB4* | 5’-GCAGCTTCCAAATCACAGAGG-3’ (sense) |
|  | 5’-CCAGATCATCGGACATGGAGTT-3’ (antisense) |
| h*ITGB5* | 5’-TCTCGGTGTGATCTGAGGG-3’ (sense) |
|  | 5’-TGGCGAACCTGTAGCTGGA-3’ (antisense) |
| h*ITGB6* | 5’-TCCATCTGGAGTTGGCGAAAG-3’ (sense) |
|  | 5’-TCTGTCTGCCTACACTGAGAG-3’ (antisense) |
| h*ITGB7* | 5’-AGAATGGCGGAATCCTCACCT-3’ (sense) |
|  | 5’-TGAAGTTCAGTTGCTTGCACC-3’ (antisense) |
| h*ITGB8* | 5’-ACCAGGAGAAGTGTCTATCCAG-3’ (sense) |
|  | 5’-CCAAGACGAAAGTCACGGGA-3’ (antisense) |
| h*SOX2* | 5’-GCCGAGTGGAAACTTTTGTCG-3’ (sense) |
|  | 5’-GGCAGCGTGTACTTATCCTTCT-3’ (antisense) |
| h*NANOG* | 5’-TCCCGAGAAAAGATTAGTCAGCA -3’ (sense) |
|  | 5’-AGTGGGGCACCTGTTTAACTT-3’ (antisense) |
| h*NESTIN* | 5’-CTGCTACCCTTGAGACACCTG-3’ (sense) |
|  | 5’-GGGCTCTGATCTCTGCATCTAC-3’ (antisense) |
| h*MAFG* | 5’-GTGAGAGCGCCTGCTCG-3’ (sense) |
|  | 5’- GACATGGTCACCAGCTCCTC-3’ (antisense) |
| h*RhoA* | 5’-AGCCTGTGGAAAGACATGCTT-3’ (sense) |
|  | 5’-TCAAACACTGTGGGCACATAC-3’ (antisense) |
| h*Talin* | 5’-GACGATGCAGTTTGAGCCG-3’ (sense) |
|  | 5’-GGGTCATCATCTGACAGAAAGAG-3’ (antisense) |
| h*Kindlin2* | 5’-TCTGACCATGCTCTCTGGTG-3’ (sense) |
|  | 5’-AGTTCTTCGGGGTGTCTGATA-3’ (antisense) |
| h*PTP-PEST* | 5’-AGTTGCCTTGTTGAAGGGGAT-3’ (sense) |
|  | 5’-AGAAGGTGTCAAGATGGGTGG-3’ (antisense) |

**Table S6 The information of SGRNA sequences used for CRISPR-Cas 9**

| CRISPR-Cas 9 Primers | |
| --- | --- |
| SGCTRL (mice) | CACCGGGGCGAGGAGCTGTTCACCG (sense) |
|  | AAACCGGTGAACAGCTCC TCGCCCC (antisense) |
| *ITGB8*-SGRNA1 (mice) | AATCAATACCCAGGTGACAC (sense) |
|  | GTGTCACCTGGGTATTGATT (antisense) |
| *ITGB8*-SGRNA2 (mice) | ACGTTACAACATGCACAGAC (sense) |
|  | GTCTGTGCATGTTGTAACGT (antisense) |
| *RhoGDI*-SGRNA1 (mice) | GGAGATCCAGGAACTGGACA (sense) |
|  | TGTCCAGTTCCTGGATCTCC (antisense) |
| *RhoGDI*-SGRNA2 (mice) | GGGCCTCCTTGTACTTTCGG (sense) |
|  | CCGAAAGTACAAGGAGGCCC (antisense) |
| *YAP*-SGRNA1 (mice) | CCGGCCGGCCACCAGGTCGT (sense) |
|  | ACGACCTGGTGGCCGGCCGG (antisense) |
| *YAP*-SGRNA2 (mice) | CCAGGTCGTGCACGTCCGCG (sense) |
|  | CGCGGACGTGCACGACCTGG (antisense) |
| *MAFG*-SGRNA1 (mice) | TGGCACCAGCTTGACCGACG (sense) |
|  | CGTCGGTCAAGCTGGTGCCA (antisense) |
| *MAFG*-SGRNA2 (mice) | GAGTTGAACCAGCACCTGCG (sense) |
|  | CGCAGGTGCTGGTTCAACTC (antisense) |
| *LATS1*-SGRNA1 (mice) | CAGAAGGATATAGACAAATG (sense) |
|  | CATTTGTCTATATCCTTCTG (antisense) |
| *LATS1*-SGRNA2 (mice) | AGTATAGTTAC TGGCAGGAA (sense) |
|  | TTCCTGCCAGTAACTATACT (antisense) |
| *LATS2*-SGRNA1 (mice) | TTTCCAGAGTAAGTTGTGGC (sense) |
|  | GCCACAACTTACT CTGGAAA (antisense) |
| *LATS2*-SGRNA2 (mice) | GAATCTCTTGCAATCGCTGC (sense) |
|  | GCAGCGATTGCAAGAGATTC (antisense) |
| SGCTRL (human) | CACCGGGGCGAGGAGCTGTTCACCG (sense) |
|  | AAACCGGTGAACAGCTCC TCGCCCC (antisense) |
| *ITGB8*-SGRNA1 (human) | TGCCTGCAAAACGACCGGCG (sense) |
|  | CGCCGGTCGTTTTGCAGGCA (antisense) |
| *ITGB8*-SGRNA2 (human) | TTGCAGGCAGACAAATGCAG (sense) |
|  | CTGCATTTGTCTGCCTGCAA (antisense) |
| *RhoGDI*-SGRNA1 (human) | CCGGCCCAGAAGAGCATCCA (sense) |
|  | TGGATGCTCTTCTGGGCCGG (antisense) |
| *RhoGDI*-SGRNA2 (human) | TGTC CAGCTCCTGGATCTCC (sense) |
|  | TGCACACAGTGTAAGCCAAG (antisense) |
| *YAP*-SGRNA1 (human) | TCGAACATGCTGTGGAGTCA (sense) |
|  | TGACTCCAC AGCATGTTCGA (antisense) |
| *YAP*-SGRNA2 (human) | CAACTGCAGAGAAGCTG GAG (sense) |
|  | CTCCAGCTTCTCTGCAGTTG (antisense) |
| *MAFG*-SGRNA1 (human) | TGAGGAGCTGGTGACCATGT (sense) |
|  | ACATGGTCACCAGCTCCT CA (antisense) |
| *MAFG*-SGRNA2 (human) | CGGCGCACGCTCAAGAACCG (sense) |
|  | CGGTTCTTGAGCGTGCGCCG (antisense) |
| *LATS1*-SGRNA1 (human) | CAGAA GGATATAGACAAATG (sense) |
|  | CATTTGTCTATATCCTTCTG (antisense) |
| *LATS1*-SGRNA2 (human) | AGTATAGTTACTGGCAGGAA (sense) |
|  | TTCCTGCC AGTAACTATACT (antisense) |
| *LATS2*-SGRNA1 (human) | GTCGTGGCAGGAAAA GTCTT (sense) |
|  | AAGACTTTTCCTGCCACGAC (antisense) |
| *LATS2*-SGRNA2 (human) | GAATCTCTTGCAGTCGCTGC (sense) |
|  | GCAGCGACTGCAAGAGAT TC (antisense) |

**Table S7 The information of siRNA sequences used for gene silencing**

| siRNAs | |
| --- | --- |
| Human siRhoA#1 | 5-GAAGGATCTTCGGAATGAT-3 |
| Human siRhoA#2 | 5- GACCAAAGATGGAGTGAGA-3 |
| mouse siRhoA#1 | 5- GGTAAGACATGCTTGC TCA-3 |
| mouse siRhoA#2 | 5- GGAGCTTGTGGTAAGACAT-3 |
| human siTalin1#1 | 5-CAGCGACTTTGGGCTCTTTCTGT-3 |
| human siTalin1#2 | 5- ACCAATCATGAT GAATATTCATT-3 |
| mouse siTalin1#1 | 5- ACCATTTGTGCCCGAATTGGTAT-3 |
| mouse siTalin1#2 | 5-TCCTAGTCAAGGAAAAGATGAAG-3 |
| human siKindlin2#1 | 5-GTGGAGAAACTCGATGTAAAAAA-3 |
| human siKindlin2#2 | 5-TGGAGAAAC TCGATGTAAAAAAA-3 |
| mouse siKindlin2#1 | 5-GTGGAAAAACTCGATGTCAA AAA-3 |
| mouse siKindlin2#2 | 5-TGGAAAAACTCGATGTCAAAAAA-3 |
| human siPTP-PEST#1 | 5-GGGGGTATTGAGCGGTTAAGAAG -3 |
| human siPTP-PEST#2 | 5-GGGGTATTGAGCGGTTAAGAAGA -3 |
| mouse siPTP-PEST#1 | 5-GAGAAGAT TGTCTACCAAATATA -3 |
| mouse siPTP-PEST#2 | 5-TACCAAATATAGAACAGAA AAGA -3 |
| Negative control | 5-GGCUCUAGAAAAGCCUAUGCTT-3 |
